# Model Mechanism for Lipid Uptake by the Human STARD2/PC-TP Phosphatidylcholine Transfer Protein

**DOI:** 10.1021/acs.jpclett.4c01743

**Published:** 2024-08-06

**Authors:** Reza Talandashti, Mahmoud Moqadam, Nathalie Reuter

**Affiliations:** † Department of Chemistry, University of Bergen, Bergen 5020, Norway; ‡ Computational Biology Unit, Department of Informatics, University of Bergen, Bergen 5020, Norway

## Abstract

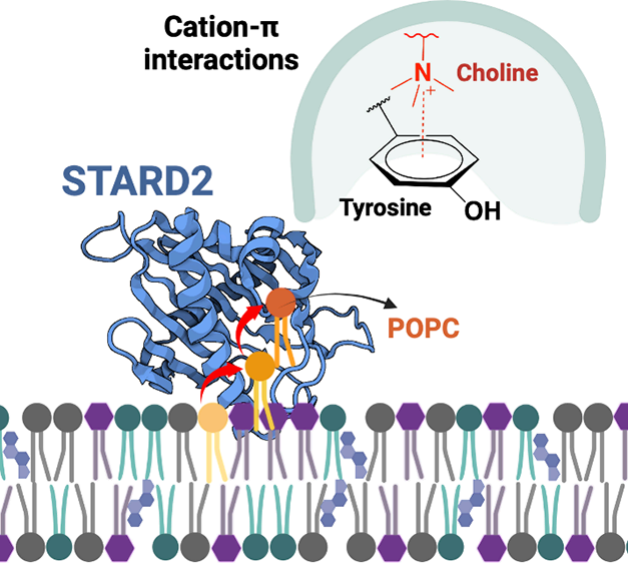

The human StAR-related lipid transfer domain protein 2 (STARD2), also known as phosphatidylcholine (PC) transfer protein, is a single-domain lipid transfer protein thought to transfer PC lipids between intracellular membranes. We performed extensive μs-long molecular dynamics simulations of STARD2 of its apo and holo forms in the presence or absence of complex lipid bilayers. The simulations in water reveal ligand-dependent conformational changes. In the 2 μs-long simulations of apo STARD2 in the presence of a lipid bilayer, we observed spontaneous reproducible PC lipid uptake into the protein hydrophobic cavity. We propose that the lipid extraction mechanism involves one to two metastable states stabilized by choline-tyrosine or choline-tryptophane cation-π interactions. Using free energy perturbation, we evaluate that PC-tyrosine cation-π interactions contribute 1.8 and 2.5 kcal/mol to the affinity of a PC-STARD2 metastable state, thus potentially providing a significant decrease of the energy barrier required for lipid desorption.

Lipid transfer proteins (LTPs) are a large group of proteins that transfer lipids between intracellular membranes and are found across evolutionarily distant organisms.^1,2^ LTPs bind to their *donor* membrane from which they selectively extract their *cargo* lipid which they transfer to -and release into- the *acceptor* membrane. One major group of LTPs in human is the steroidogenic acute regulatory protein (StAR)-related lipid-transfer (StART) domain, which consists of 15 domains.^1,3^ The 15 proteins share a common helix-grip fold surrounding a large hydrophobic cavity holding the cargo lipid. StAR-related lipid transfer domain protein 2 (STARD2), also known as phosphatidylcholine transfer protein (PC-TP), is a single-domain protein in humans that has demonstrated the ability to extract phosphatidylcholine (PC) lipids from vesicles and microsomes, with a preference for PC lipids containing polyunsaturated tails.^4−7^ STARD2 is thought to extract PC synthesized in the endoplasmic reticulum (ER) and transfer it to the mitochondria^8^ or plasma membrane.^9^ It has also been suggested that STARD2 is a PC sensor involved in metabolic regulation.^10^ The available X-ray structures of STARD2 in complex with PC lipids show the choline headgroup sitting in an aromatic cage on one side of the hydrophobic cavity, and formed by W101, Y114, Y116 and Y155 (Figure 1 and S1).^4−6^ Yet the mechanisms of PC release and extraction by STARD2 remain unknown.

**Figure 1:**
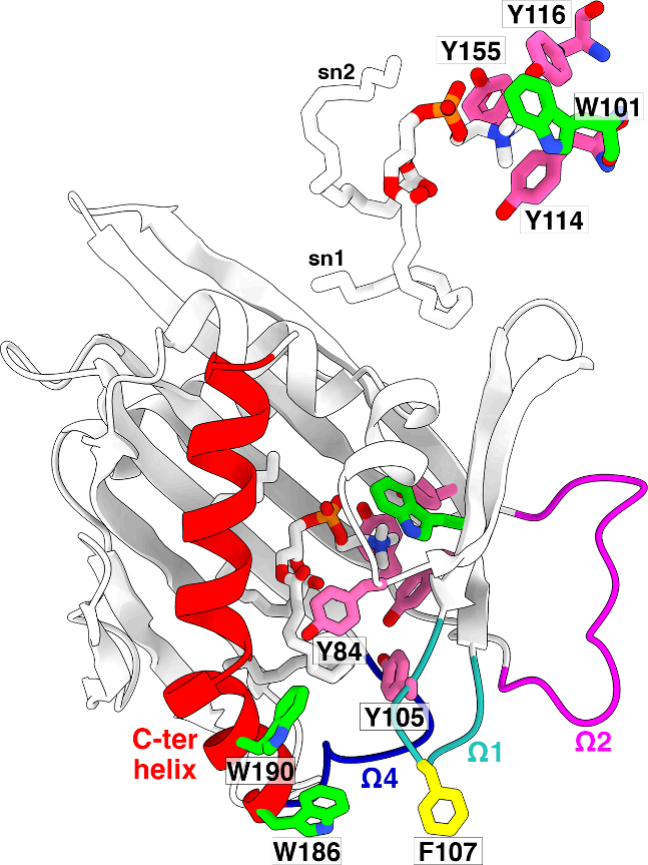
**X-ray structure of STARD2**. The structure of STARD2 in complex with dilinoleoyl-*sn*-glycero-3-phosphocholine (DLPC) (PDB ID: 1LN1) is depicted in a cartoon model, colored predominantly in white, except for the C-terminal helix (red) and the three loops Ω1 (cyan), Ω2 (magenta) and Ω4 (blue). Aromatic amino acids surrounding the choline moiety of phosphatidylcholine, as well as aromatic residues of the gate region, are represented in a stick model and colored in green (Trp), yellow (Phe), and pink (Tyr). The DLPC lipid is shown as sticks and colored by atom type (C: white, O: red, P: orange, N: blue).

Our understanding of the mechanisms by which LTPs in general overcome the high energy barrier of lipid extraction (or release) from (or to) a well-packed lipid bilayer, is very limited so far. The spatial resolution and time scales of these processes render them challenging to study using experimental techniques. Molecular dynamics (MD) simulations can be used to map interactions between the LTP and its cargo, between the cargo and membrane and between the LTP and membrane, to understand the balance between these interactions and how it effectuates lipid extraction or release. For instance, molecular simulations of the ceramide-1-phosphate transfer protein (CPTP) showed that the uptake and release of ceramide-1-phosphate by CPTP is facilitated by conformational changes of the CPTP coupled with the disruption of lipids packing below the protein, and the creation of protein-cargo hydrophobic contacts.^11^ In simulations of the ceramide transfer protein CERT (STARD11) with lipid bilayers we also observed that the opening of the gate is concomitant to changes in lipid tails packing. In addition, the intercalation of a single phosphatidylcholine lipid in the cavity disrupts the LTP-cargo interactions, and facilitates the release of the ceramide cargo through the polar membrane interface.^12^ It is worth noting that in STARD11, like for STARD2 but unlike for CPTP, the lipid cargo is loaded head first.

The abundance of aromatic residues in the hydrophobic cavity and the gate region of STARD2 (Figure 1), along with the nature of the choline headgroup, suggests the potential involvement of cation-π interactions in the membrane-binding or PC uptake mechanisms by STARD2. Cation-π interactions are widespread noncovalent interactions that are important in protein folding and stability,^13−15^ ligand recognition,^16^ and recognition of choline-containing lipids by peripheral membrane proteins.^17−22^ The *B.thuringiensis* phosphatidylinositol-specific phospholipase C (*Bt*PI–PLC) relies on multiple surface-exposed tyrosines engaging in cation−π interactions to selectively recognize choline-containing lipids.^17,23−25^ Spider venom GDPD-like phospholipases D recognize choline-containing lipids thanks to an evolutionary conserved aromatic cage.^26^ We earlier estimated that choline-tyrosine and choline-tryptophane cation−π interactions contribute between 2.5 and 3.5 kcal/mol to the membrane affinity of *Bt*PI–PLC or of the snake venom *Naja naja atra* phospholipase A2.^27^

In what follows we report the results of our investigations of the role of the aromatic amino acids present at the surface of STARD2 in the uptake of phosphatidylcholine-containing lipids. We conducted multiple μs-long all atoms MD simulations of STARD2 in the presence of membrane models. For that purpose, we used the NAMD3^28^ simulation package and the CHARMM36 force field,^29,30^ with hydrogen mass repartitioning (HMR)^31,32^ to lower the computational cost of the simulations.

We first built a model of the apo form of STARD2 by removing the DLPC lipid from a holo X-ray structure (PDB ID: 1LN1).^5^ There are indeed no structures of STARD2 in its apo form. After careful minimization and equilibration of the protein in an aqueous solution to allow for hydration of the cavity, we simulated it for 1 μs (see the SI for details). The simulations of the holo form, which were performed as control simulations, are stable and STARD2 remains close to the X-ray structure (average RMSD < 2 Å) (Figure S2A). Moreover, the spatial configuration of the lipids inside the cavity does not deviate significantly from the X-ray structure (Figure S3). In the simulations of apo STARD2, we observe a change of the orientation of W101 modifying the aromatic cage (Figure S2C and S2D) which is likely to be the consequence of the removal of the bound lipid from the holo X-ray structure. Most importantly the apo STARD2 undergoes notable conformational changes (average RMSD of 2.6 Å) with a bending of the Ω3 loop and an inward displacement of the C-ter helix (Figure S2A-B). Our simulations thus show ligand-dependent conformational changes which might form the basis for the observed differential STARD2-PPARδ recognition, suggesting that the C-ter helix forms part of the STARD2-PPARδ interface. Druzak et al.^4^ indeed showed that mutations in the PC binding site, which reduce STARD2-PC binding, resulted in decreased STARD2-PPARδ interactions and suggested a modulation of this interaction by the STARD2 ligands. Kang et al. also suggested modulation of the STARD2-Them2 interactions by conformational changes upon PC binding.^7^

We next investigated the membrane selectivity of STARD2. The ability to distinguish between donor and acceptor membranes varies widely among LTPs. Some are highly sensitive to lipid composition, such as Osh4^33,34^ and STARD4 which show specificity toward phosphatidylinositol bisphosphate (PIP2) lipids,^35,36^ while other LTPs show low or no sensitivity to lipid compositions such as the CERT START domain (STARD11).^12^ We performed simulations of apo STARD2 in the presence of an ER bilayer model, and of its holo form in the presence of the outer mitochondrial membrane (OMM) (see SI for the lipid composition). To correct for the change of orientation of W101 observed in the apo-water simulation, we applied a dihedral restraint on the W101 χ1 and χ2 angles to maintain its experimental conformation and facilitate the lipid uptake (see Methods section for detailed protocols). STARD2 binds to the lipid bilayers within the first 500 ns of simulations (Figure S4). For both apo-ER and holo-OMM the membrane binding orientation and insertion depth of STARD2 are comparable (Figure 2 and S5–6). The loops Ω1 and Ω4, and the N-terminus end of the C-ter helix are inserted in the bilayer interface.

**Figure 2:**
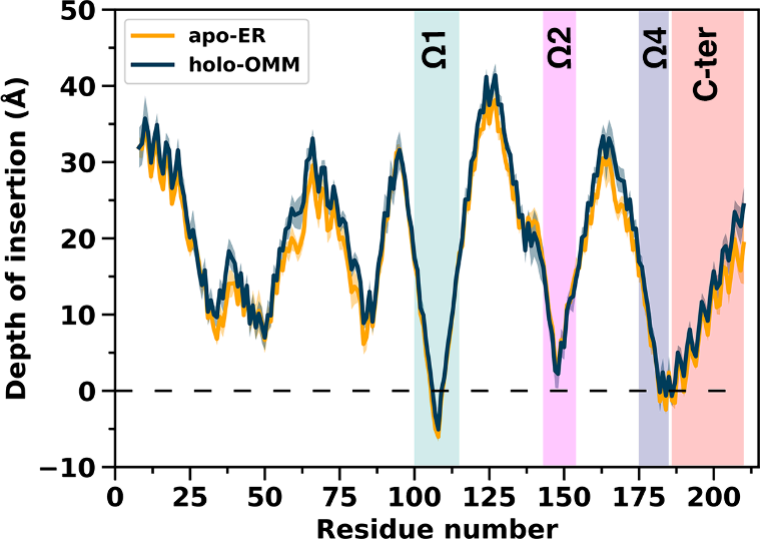
**Depth of insertion of apo STARD2 on the ER bilayer and holo STARD2 on the OMM bilayer**. The distance between the average phosphate plane (dashed line, Y = 0) and the β carbon of each residue is plotted. The values for the apo-ER system are an average over the six replicas, while for the holo-OMM systems they are averages over the three replicas of holo_DLPC-OMM, of holo_PLPC-OMM and of holo_PAPC-OMM (9 simulations in total). The shaded area represents the standard deviation for each curve.

Amino acids S110 and R112 (Ω1) engage in long-lasting hydrogen bonds with lipid phosphate groups; the occupancy of these interactions is between 75% and 97% in the apo and holo simulations (Table 1). R43 (β2), Q182 (Ω4) and S185 (C-ter) engage in strong hydrogen bonds with the lipids, with occupancies varying from 43% to 77% in the four simulation systems. Furthermore, many amino acids from the Ω1 loop (Y84, P106, F107, P108, and M109), the Ω4 loop (Q182), and the C-ter helix (I83, P184, W186, L187, I188, W190, and A191) establish hydrophobic contacts with the lipid tails further showing that their insertion at the interface is quite deep (Table 1, Figure S6). The occupancies for the C-ter helix tend to be higher in the apo than in the holo simulations reflecting that the conformational change in the apo form results in more interactions between the helix and the bilayer (Table 1).

**Table 1:** Hydrogen Bonds and Hydrophobic Contacts between STARD2 Amino Acids and Bilayer Lipids

		apo-ER	holo_DLPC-OMM	holo_PLPC-OMM	holo_PAPC-OMM
		Hydrogen bonds occupancy^a^ (%)			
β2	R43^c^	47.5	42.7	51.8	40.8
Ω1	S110^d^	74.5	94.4	81.1	96.0
	R112^d^	76.7	93.8	85.1	96.8
Ω4	Q182^d^	58.3	57.3	49.3	58.9
C-ter helix	S185^d^	63.5	55.4	76.4	42.6
	W186^d^	48.4	27.5	38.6	22.3
	W190^d^	30.9	14.9	22.5	12
	K193^d^	47.3	18.8	22.3	22.2

		Hydrophobic contacts^b^			
Ω1	Y84	0.5	0.13	0.1	0
	P106	1.4	0.6	0.8	0.3
	F107	2.3	1.5	2.1	2.6
	P108	1.9	1.3	1.4	1.6
	M109	0.9	0.5	0.5	0.6
Ω4	Q182	0.8	0.7	0.6	0.5
C-ter helix	I183	0.8	0.3	0.2	0.2
	P184	1.9	0.5	1.3	0.6
	W186	2	2.1	1.5	1.2
	L187	1.3	0.35	0.5	0
	I188	0.6	0	0	0
	W190	1.4	1.1	1.1	0.9
	A191	1.3	0	0.3	0

a Average occupancy of hydrogen bonds over all replicas, calculated over the last 500 ns of trajectories and reported if the value is above 30% in at least one system, and present in all replicas.

b Average number of hydrophobic contacts per trajectory frame over all replicas for each system, calculated during the last 500 ns of simulations, and reported if greater than 0.5.

c Hydrogen bond established between amino acid side chain and anionic lipid headgroup (POPS, POPI).

d Hydrogen bond established between amino acid side chain and lipid phosphate.

The simulations of holo STARD2 do not reveal significant changes in the positions of the headgroup of the bound lipids but we do observe some changes in the positions of their acyl chains after STARD2 becomes anchored at the lipid bilayer. In two replicas of the holo_PLPC-OMM simulations, the sn-1 lipid tail (saturated) is inserted into the lipid bilayer, while in the third replica, both the sn-1 and sn-2 tails are intercalated between the tails of bilayer lipids (Figure S7B). In the holo_DLPC-OMM simulations, insertion of the sn-1 tail into the bilayer was observed in only one replica (Figure S7A). Similarly, in the holo_PAPC-OMM simulations, sn-1 tail insertion occurred in two replicas (Figure S7C). In all simulations where tail insertion events were observed, the headgroup of the PC lipid remained in the aromatic cage, and the phosphate group remained hydrogen bonded to residues R78, Y72, and Q157. No spontaneous release is observed unlike what we observed in CERT/STARD11,^11^ indicating that the simulations do not sample interactions or events that would lower the energy barrier for the release of the lipid.

In what follows we focus on the simulations of apo STARD2 on the ER bilayer, and on the model for the POPC uptake mechanism emerging from those simulations. The inventory of cation-pi interactions during the 2 μs-long simulations shows that 13 aromatic amino acids engage in interactions with choline groups but with only low to moderate occupancies (24% at most on average, Table S3) compared to what we earlier observed for other peripheral membrane proteins (up to 95%).^23,26,37^ These interactions are thus unlikely to strongly contribute to the overall STARD2-bilayer affinity. Also, several of the involved aromatics are located between 9 to 15 Å above the average phosphate plane, including the four amino acids from the aromatic cage (W101, Y114, Y116, Y155) (Figure 1) and Y84 lining the walls of the cavity. These locations suggested another important role for the function of STARD2 which was revealed by visual inspections of the trajectories (Figure 3) and confirmed by time series of the cation-π interactions by W186, W190, Y84, Y105 and by the aromatic cage (Figure 4). In two of the six replicas (Rep2, Rep3) we observe spontaneous uptake of a POPC lipid from the bilayer and to the aromatic cage, progressing along the cavity through cation-pi interactions (Figure S8). The final conformation of the lipid headgroup is comparable to that of the holo X-ray structure but the tails remain in an extended form unlike in the X-ray structure (Figure S9), indicating that we only capture parts of the uptake mechanism. The uptake proceeds in three steps illustrated on Figure 3.

**Figure 3:**
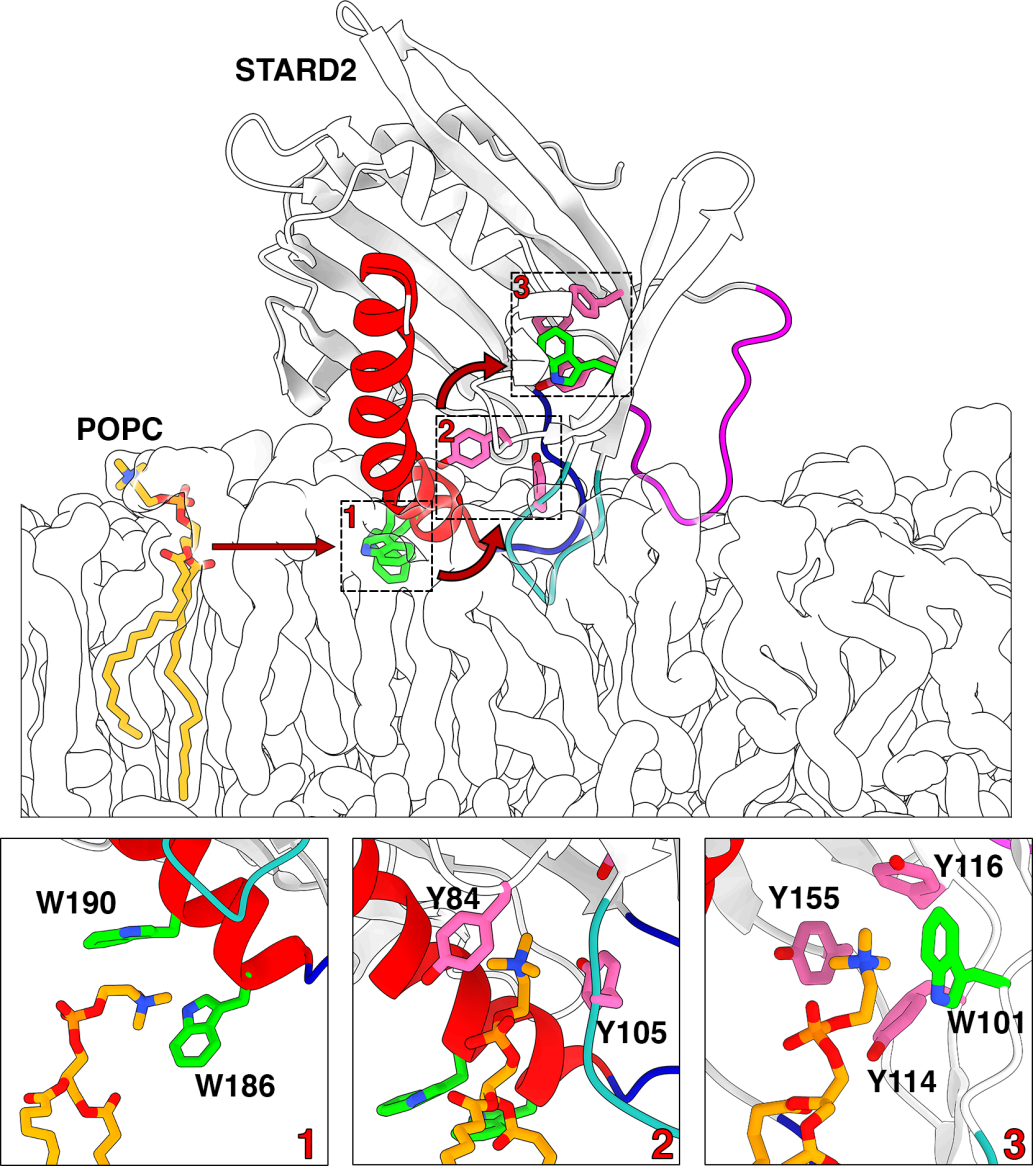
**Proposed POPC uptake mechanism**. The top panel illustrates the trajectory of a POPC lipid from the ER bilayer to site 1 (W186, W190), via site 2(Y84, Y105) and to the aromatic cage (site3, W101, Y114, Y116, Y155). The bottom panels show the choline-aromatics cation-π interactions at each of the three sites. The POPC and aromatics are shown with sticks (see Figure 2 for color scheme).

**Figure 4:**
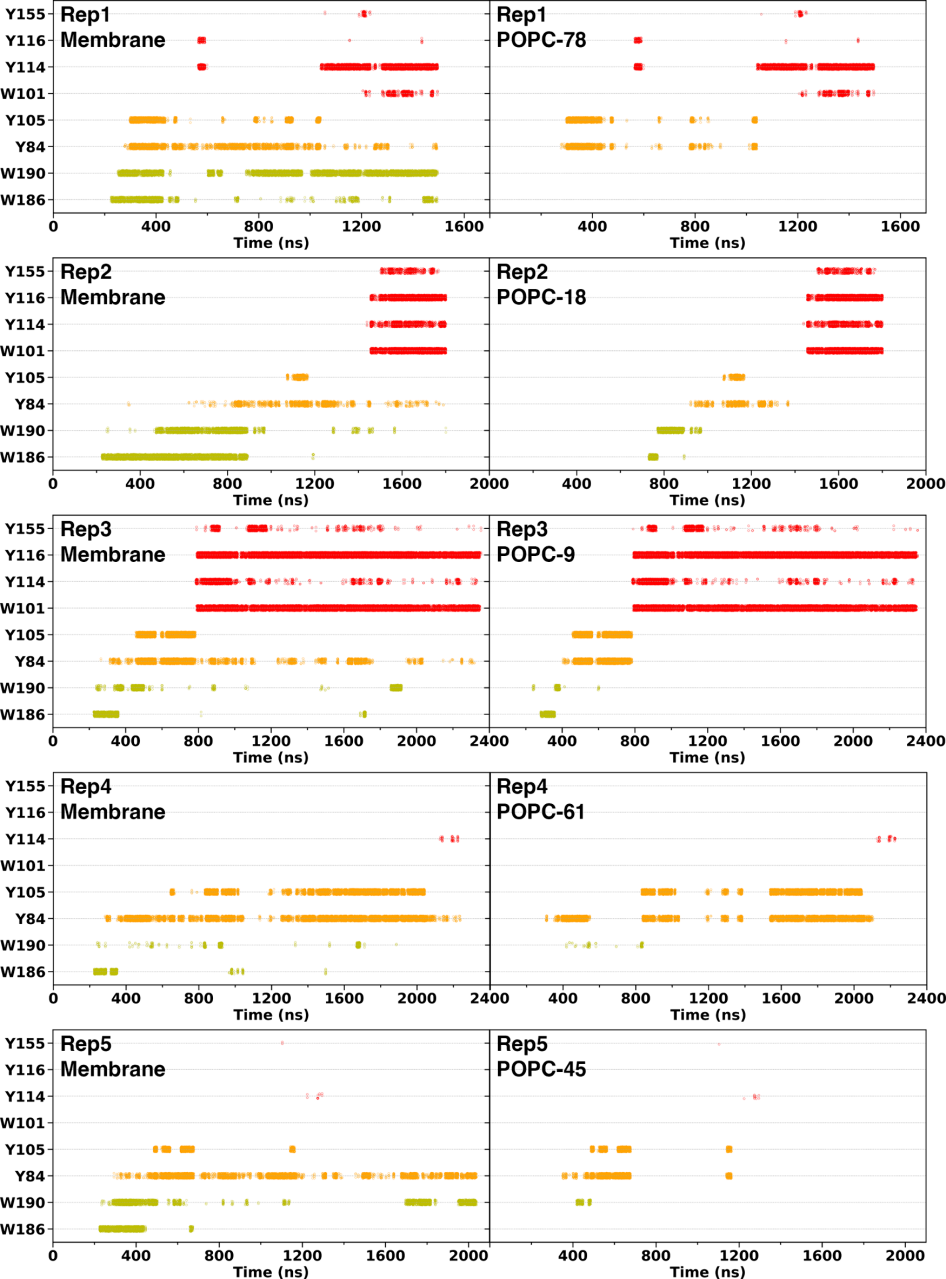
**Choline-aromatics cation-π interactions across STARD2-ER simulation replicates**. The left panels show cation-π interactions between the selected residues at site 1 (W186 and W190), site 2 (Y84 and Y105), and site 3 (W101, Y114, Y116, and Y155) with all membrane lipids, while the right panels focus on cation-π interactions between the same residues and only the POPC bound in the cavity.

First the choline headgroup interacts with residues W186 and W190 located on the N-terminus part of the C-terminal helix (site 1). In the second step, the choline group interacts with Y84 and Y105 situated on the cavity wall (site 2) and in the third step, the PC transitions from site 2 to the aromatic cage (site 3) (Figure 3). This is also confirmed by the time series of cation-π interactions shown on Figure 4. In one of replicas (Rep1), we also observe spontaneous uptake, but the POPC lipid proceeds directly to site 2 before moving up toward the cage and engaging in cation-pi interactions. Yet the headgroup does not reach a stable position in the cage. During the uptake, site 1 is occupied by another POPC lipid interacting in particular with W190. In two other replicas (Rep4 and Rep5) a POPC lipid is taken up to sites 1 and 2 successively but does not reach a stable position in the aromatic cage within the 2 μs-long simulations. In the last of the six replicas (Rep6) we observe interactions of a POPC with site 1 but no stable interactions with site 2 and no extraction from the bilayer (Figure S11A). Note that we used orientational restraints on W101 in the apo-ER simulations as explained above. In the absence of restraints, the flipped W101 conformation led to only partial lipid uptake up to site 2 (see Figure S10) as the indole is placed at the center of the aromatic cage and is not displaced by the POPC headgroup (Figure S10B).

Overall, our simulations suggest the existence of one to two intermediates in the lipid uptake mechanism. These intermediates are stabilized by choline-tyrosine and choline-tryptophane cation-π interactions, which might partly compensate for the cost of desorbing a POPC lipid from the bilayer. As a control, we conducted simulations of a mutant where tyrosines in site 2 are replaced by serines, to conserve their polar character but prevent cation-pi interactions (Y84S/Y105S). The mutant binds quickly to the bilayer (Figure S4) in the same orientation and at the same depth (Figure S5) as the wild type but we do not observe PC uptake in any of the three replicates. We do observe cation-π interactions between POPC lipids and site 1, but no interactions with site 2 (Figure S11B). This confirms the pivotal role of the tyrosines in site 2 for uptake of POPC to the aromatic cage.

We propose that the cation-π interactions play the same role as the protein-cargo hydrophobic contacts described in the work of Rogers et al., where it was estimated that the ceramide transfer protein reduces the free energy of desorption by 2.5 kcal/mol.^11^ We here assume that the two tryptophanes in site 1 (W186, W190) contribute 3 to 3.5 kcal/mol each, based on earlier reported computational and experimental data.^27^ For interfacial choline-tyrosine cation-π interactions we earlier reported contributions to protein–membrane affinity of 1.5–2.5 kcal/mol. However, these values might not be directly transferable to site 2 since Y84 and Y105 are located away from the membrane interface. We therefore calculated the contributions of the POPC-Y84 and POPC-Y105 cation-π interactions to the affinity between STARD2 and the POPC lipid inserted in the cavity. We used free energy perturbations (FEP) following the cycle shown on Figure 5. The calculations yield a ΔΔ*G*_binding_(POPC) for Y84 of 1.8 kcal/mol, and 2.5 kcal for Y105, which are within the range of values for interfacial tyrosines (Table 2).

**Figure 5:**
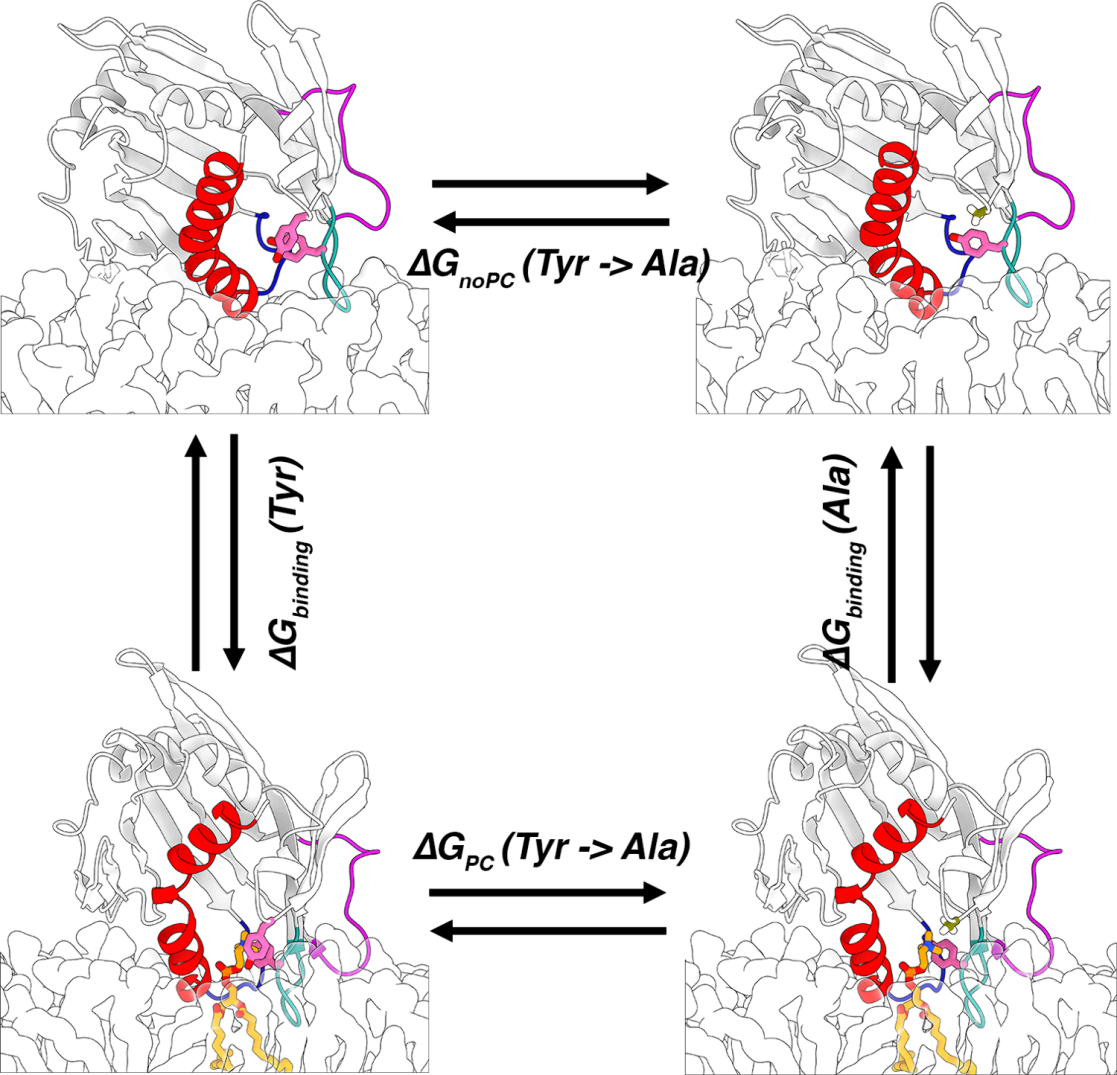
**Thermodynamic cycle for FEP calculations**. The cycle is illustrated with the substitution of Tyr84 (pink sticks) by alanine (olive stick) in the membrane-bound form of STARD2 (cartoons) in the absence *(ΔG*_*noPC*_*(Tyr → Ala))* and presence *(ΔG*_*PC*_*(Tyr → Ala))* of a bound POPC (orange sticks).

**Table 2:** Contribution of Y84 and Y105 to POPC-STARD2 Binding Free Energy from FEP Calculations^a^

Mutation		Forward (kcal/mol)	Backward (kcal/mol)	BAR (kcal/mol)	ΔΔ*G*_binding_ (kcal/mol)
Y84A	PC	12.38	–11.29	11.98 ± 0.12	1.81
	noPC	10.43	–9.62	10.17 ± 0.08	
Y105A	PC	14.89	–12.15	13.34 ± 0.18	2.54
	noPC	9.96	–11.72	10.79 ± 0.08	

a Transformation of each tyrosine to alanine in the absence and presence of POPC from forward and backward simulations and the corresponding BAR estimate.

The barrier for desorption of a POPC lipid from a POPC bilayer has been estimated to be around 14 kcal/mol, and varying between 12.6 and 17.3 kcal/mol for extraction of POPG and POPE from POPG and POPE bilayers, respectively.^38^ The predicted contributions of tyrosines in site 2 and tryptophanes in site 1 to POPC binding range from 1.8 to 3.5 kcal/mol each, which is equivalent (for each aromatic) to 13 to 25% of the reported barrier for desorption of POPC. It is important to note that we cannot assume that these contributions are additive, as they are not all coexisting at a given time. Effects such as protein–lipid hydrophobic contacts (as in Rogers et al.^11^) and disruption of lipid packing by STARD2 would provide additional compensating contributions. We therefore propose that the uptake mechanism involves 1–2 metastable intermediates stabilized by POPC-STARD2 cation-π interactions which facilitate POPC extraction by STARD2. The full uptake mechanism of the whole lipid in the hydrophobic cavity is likely to be more complex. Indeed our proposed model does not account for the full insertion of the acyl chains and this is most likely due to insufficient sampling. STARD2 might also undergo larger conformational changes that are not captured in μs-long simulations.

Using extensive equilibrium MD simulations, we shed light on several aspects of STARD2 function. First, the cargo-dependent conformational change observed in our simulations provides a possible explanation for the experimentally observed change in STARD2-PPARδ interactions pointing at the C-ter helix as a possible region of the protein–protein interface. Second, we do not observe differences in STARD2 binding to the OMM and ER bilayer models, and hence no indication of STARD2 binding selectively to one or the other membranes, within the limits of our models. Third, and most importantly we propose a model for the mechanism by which STARD2 extracts PC lipids from membranes. This mechanism relies on metastable intermediate states, stabilized by cation-π interactions between the choline headgroup of the extracted PC and conserved tyrosines and tryptophanes. Rigorous evaluation of their energetic contribution show that it would significantly reduce the energetic cost of desorbing a POPC lipid from its membrane. In the same time frame as the ones displaying the POPC uptake (2 μs) we do not observe the full release of the lipid in the membrane-bound holo STARD2, indicating that a different mechanism is at play. It is likely that an additional event is needed to weaken the strong interactions maintaining the choline group in the aromatic cage.

The results presented here and obtained through extensive equilibrium MD simulations and free energy calculations advance our understanding of choline-containing lipid extraction by STARD2. They also provide new hypotheses experimentally testable such as the expected inhibiting effect that substituting Y84 and Y105 into serine would have on STARD2 transfer activity. The proposed model is also a stepping stone for future computational investigations, for example using enhanced-sampling methods, to obtain a more comprehensive picture of the energetics of the whole uptake mechanism process including full insertion of the acyl chains. Future work focusing on other LTPs selectively transferring choline-containing lipids might benefit from investigating the transferability of the proposed mechanism by mapping the presence of aromatic amino acids in relevant regions of LTP structures.

## Computational Methods

The three structures of holo STARD2 with DLPC, PLPC and PAPC were extracted from the RCSB Protein Data Bank with respective PDB IDs 1LN1,^5^ 1LN3,^5^ and 7U9D.^4^ The model of apo STARD2 was built from the X-ray structure of STARD2:DLPC by removing the lipid. All systems were prepared for simulations with CHARMM-GUI,^39,40^ including solvation and addition of neutralizing ions. The lipid composition of the OMM bilayer model closely resembles that of the ER, with the exception of the absence of cholesterol and the presence of cardiolipin instead of ceramide. All reported simulations were conducted with the NAMD3 package^28^ and employing the CHARMM36m force field^29,30^ along with its CHARMM-WYF^41,42^ extension for cation-π interactions. We applied hydrogen mass repartitioning (HMR)^31,32^ to the protein, cargo lipid, and lipid bilayers in production runs and a 4 fs integration time step was used. Temperature was maintained at 310 K using Langevin dynamics with a temperature damping coefficient of 1 ps-1, while pressure was controlled at 1 atm utilizing a Langevin piston with an oscillation period of 200 fs. We employed an alchemical approach to calculate the contribution of Y84-choline and Y105-choline π-cation interactions to the affinity of the POPC lipid for STARD2. We followed an alchemical route for transforming the Y84 and Y105 to alanine, both in the presence and absence of the PC headgroup. We achieved this by performing free energy perturbation (FEP) simulations along the horizontal directions in Figure 5. Statistical analyses of the FEP simulations were carried out by combining forward and backward simulations using the Bennett acceptance ratio (BAR)^43^ algorithm via the ParseFEP^44^ plugin in VMD. The details of all simulations and their analysis, and of the FEP simulations are provided as SI.

## Data Availability

All MD trajectories are uploaded to the Norwegian national infrastructure for research data (NIRD) and can be accessed using the following DOI: 10.11582/2024.00104.

## Supporting Information Available

Details of the computational methods and protocols, a list of simulated systems and their composition including the lipid composition of the membrane models, snapshots and analysis of trajectories from simulations of apo and holo forms of STARD2 in water, minimum protein-bilayer distances along simulation time and the depth of insertion for each amino acid, analyses and snapshots of STARD2-bilayer simulations, and occupancies and time-series of bilayer-STARD2 cation-π interactions (PDF)
Transparent Peer Review report available (PDF)
