# Supplementary material for: Model Mechanism for Lipid Uptake by the Human STARD2/PC-TP Phosphatidylcholine Transfer Protein
Source: J Phys Chem Lett. 2024 Aug 6;15(32):8287–95. doi: 10.1021/acs.jpclett.4c01743 (PMC11331517; doi:10.1021/acs.jpclett.4c01743)
Supplement: Supplementary file 1 — jz4c01743_si_001.pdf [file jz4c01743_si_001.pdf]

Supporting information for

Model mechanism for lipid uptake by the human STARD2/PC-TP phosphatidylcholine transfer protein

*Reza Talandashti [1,2], Mahmoud Moqadam[1,2] and Nathalie Reuter [1,2] **

[1] Department of Chemistry, University of Bergen, Bergen, 5020, Norway

[2] Computational Biology Unit, Department of Informatics, University of Bergen, Bergen, 5020, Norway

Table of Contents

Computational details S2
Table S1. List of simulated systems and their composition S2
Table S2. Lipid composition of the membrane models S4
Figure S1: X-ray structures of STARD2 S8
Figure S2: Simulations of apo and holo forms of STARD2 in water. S9
Figure S3. Structures of holo STARD2 from simulations in water. S10
Figure S4. Minimum protein-bilayer distances along simulation time. S11
Figure S5. Membrane-bound STARD2: depth of insertion for each amino acid. S12
Figure S6. Structures of membrane-bound apo-ER and holo-OMM STARD2. S13
Figure S7. STARD2 holo-OMM simulations: cargo tail insertion into the bilayers. S13
Table S3. Bilayer-STARD2 cation-π interactions. S14
Figure S8. Final frame of apo-ER simulation (Rep3). S15
Figure S9. Position of the extracted PC lipid from simulations and X-ray. S15
Figure S10. Cation-π interactions in apo_noRes-ER simulations. S16
Figure S11. Cation-π interactions between aromatic residues and bilayer lipids. S17
Figure S12. Snapshots from FEP simulations of the Y84A mutation. S17
References S18

Computational Details

1. General simulation protocol

All reported simulations were conducted with the NAMD3[1] package and employing the CHARMM36m force field[2,3] along with its CHARMM-WYF extension[4,5] for cation-π interactions. The TIP3P water model[6] and sodium ions were utilized to solvate and neutralize the systems, respectively. We applied hydrogen mass repartitioning (HMR)[7,8] to the protein, cargo lipid, and lipid bilayers only in production run and a 4 fs integration time step was used. A switching function was used for van der Waals interactions with a cutoff at 12 Å and a switching distance at 10 Å. The cutoff for electrostatic interactions was set to 12 Å. Temperature was maintained at 310 Kelvin using Langevin dynamics with a temperature damping coefficient of 1 ps^{-1}, while pressure was controlled at 1 atm utilizing a Langevin piston with an oscillation period of 200 fs. Bond lengths were constrained using the SHAKE algorithm, and particle mesh Ewald[9] was used for the treatment of long-range electrostatic interactions. We describe below how the different systems were prepared and the simulations protocols specific for each system.

Table S1. List of simulated systems and their composition. Each system was simulated for 2 μs (in addition to the equilibration steps).

Simulation type	Protein state	Lipid bilayer	Acronym	No. of replicas	Total no. of atoms	No. of ions
Water	apo	-	apo-water	1	38346	3 Na^+
	holo_DLPC	-	holo_DLPC-water	1	35396	3 Na^+
	holo_PLPC	-	holo_PLPC-water	1	35391	3 Na^+
	holo_PAPC	-	holo_PAPC-water	1	43295	3 Na^+
Outer mitochondrial membrane (OMM)	holo_DLPC	OMM	holo_DLPC-OMM	3	140508	53 Na^+
	holo_PLPC	OMM	holo_PLPC-OMM	3	137905	53 Na^+
	holo_PAPC	OMM	holo_PAPC-OMM	3	141952	53 Na^+
Endoplasmic reticulum membrane (ER)	apo_mut (Y84S/Y105S)	ER	apo_mut-ER	3	126216	43 Na^+
	apo_noRes	ER	apo_noRes-ER	3	124863	43 Na^+
	apo	ER	apo-ER	6	124079	43 Na^+

2. Simulations of STARD2 in water

Holo STARD2. Three structures of STARD2 were extracted from the RCSB Protein Data Bank. They correspond to complexes with polyunsaturated phosphatidylcholine-containing lipids: 1,2-dilinoleoyl-sn-glycero-3-phosphocholine (DLPC, 18:2) (pdb id: 1LN1)[10], 1-palmitoyl-2-linoleoyl-sn-glycero-3-phosphocholine (PLPC) (pdb id: 1LN3)[10], and 1-palmitoyl-2-arachidonoyl-sn-glycero-3-phosphocholine (PAPC) (pdb id: 7U9D)[11]. For each of the three structures, hydrogen atoms were added utilizing the VMD psfgen plugin (version 2.0)[12]. The pKa values of all ionizable residues were predicted employing PROPKA3[13] and none was predicted to deviate from their standard protonation state at pH 7. Histidine residues were protonated on their δ nitrogen, guided by visual examination of each histidine residue and their nearby amino acids to identify potential hydrogen bond donor or acceptor atoms adjacent to the δ and ε nitrogens. The systems were then prepared for simulations with CHARMM-GUI[14,15], including solvation and addition of neutralizing ions. The same simulation protocol was used for each of the three systems: energy minimization using conjugate gradients (CG, 10,000 steps), equilibration in the canonical (NVT) ensemble for 10 ns with harmonic positional restraints on all protein and lipid atoms except hydrogens, 1 μs simulation in the isothermal-isobaric (NPT) ensemble employing a 4 fs integration time step without any positional restraints.

Apo STARD2. As no experimental structure of apo STARD2 is available in the PDB, we built a model of STARD2 from the X-ray structure of holo STARD2 (PDB ID: 1LN1) by removing the DLPC lipid from the protein cavity. The system was then built using the same protocol as for holo STARD2 using CHARMM-GUI[14,15]. The simulation protocol was slightly different from that used for holo STARD2 in order to carefully relax the model of the apo protein. First, the system was minimized with CG (10,000 steps), followed by one equilibration in the NVT ensemble for 10 ns and four equilibrations in the NPT ensemble, for 5 ns each, and with decreasing force constant for the harmonic positional restraints for each of the 5 simulations (10, 5, 2.5, 1, and 0.5 kcal/mol, respectively). Finally, the system was simulated for 1 μs in the NPT ensemble without any positional restraints.

3. Preparation of lipid bilayers

The lipid compositions used to mimic endoplasmic reticulum (ER) membrane and the outer mitochondrial membrane (OMM) are provided in Table S2. For the ER bilayer we used the lipid composition we reported earlier[16] and the composition of the bilayer mimicking the OMM is based on reference[17].

Table S2. Lipid composition of the membrane models.

Outer mitochondrial membrane (OMM) model						
Lipid type	POPC	POPE	POPS	POPI	Cardiolipin	Chol
Each leaflet	50%	30%	5%	10%	5%	0%
**Endoplasmic reticulum (ER) membrane model**						
Lipid type	POPC	POPE	POPS	POPI	CER180	Chol
Each leaflet	53%	23%	5%	10%	4%	5%

Specifically, the ER bilayer model contains six different lipids, all with 1-palmitoyl-2-oleoyl chains (PO): phosphatidylcholine (PC), phosphatidylethanolamine (PE), phosphatidylserine (PS), phosphatidylinositol (PI), ceramide, and cholesterol. The lipid composition of the OMM bilayer model closely resembles that of the ER, with the exception of the absence of cholesterol and the presence of cardiolipin instead of ceramide. The ER and OMM models are symmetric lipid bilayers and contain 256 lipids each. The bilayer builder module[14] of CHARMM-GUI[5] was used to create the topology and structure files for the lipid bilayer models. Both ER membrane and OMM bilayer models were simulated using hydrogen mass repartitioning. The same protocol as employed for the protein-membrane simulations (see next section) was utilized here. In brief, following a geometry optimization using conjugate gradients (CG), the simulation protocol starts by six equilibration steps in the canonical (NVT) and then isothermal-isobaric (NPT) ensembles during which positional restraints are applied and gradually reduced at each of the six steps. Subsequently, a production run is conducted in the NPT ensemble, lasting for 200 ns with an integration step of 4 fs.

4. Simulations of holo and apo STARD2 on lipid bilayers

The starting structures of holo STARD2 are the final frames of the corresponding holo STARD2-water simulations. The mutated form of STARD2 (Y84S/Y105S) was build based from the structure of the last snapshot of the apo-water simulation via VMD Mutator plugin[12].

For every protein-bilayer system, three independent replicates were simulated, except for apo-ER simulations that 6 replicates have been performed. These replicates vary by the initial distribution of lipids in the bilayers, as the lipid bilayers were generated for each replica through a random distribution of lipids using CHARMM-GUI[14,15].

Simulations of holo STARD2 on the OMM model (holo STARD2-OMM) and of the Y84S/Y105S mutant of apo STARD2 on the ER membrane model (mutant apo STARD2-ER). The starting structures of holo and mutated STARD2 are taken from simulations in water and were positioned above the equilibrated membrane models, with the Ω1 loop directed towards the membrane to ensure a minimum protein-lipid distance of 1 nm. All protein-lipid bilayer systems were simulated with a 3.5 nm water layer above the protein and below the membrane, each for a minimum duration of 2 μs.
All simulations were conducted using HMR[7,8]. The simulation protocol involved an initial energy minimization step using conjugate gradients (10,000 steps). Subsequently, protein-membrane simulation systems underwent equilibration via two consecutive NVT simulations, each lasting for 1.25 ns, with an integration time step of 1 fs and velocity reassignment occurring every 0.5 ps. This was followed by four NPT simulations, each lasting for 5 ns, with an integration step of 2 fs. During the equilibration phase, various restraints were applied to facilitate gradual equilibration of the simulation systems. Harmonic restraints were applied to the ions and all protein atoms except for hydrogen atoms. Repulsive planar restraints were employed to prevent water molecules from infiltrating the hydrophobic region of the membrane, while planar restraints were imposed to maintain the position of the membrane's head groups along the Z-axis. These restraint forces were gradually decreased throughout the equilibration process. Following equilibration, all systems were simulated in the NPT ensemble without restraints, employing HMR and an integration time step of 4 fs. For ions, the charmm36m NBFIX corrections were employed[19].

Simulations of apo STARD2 on the ER membrane model (apo STARD2-ER). The initial frame from the apo-water simulation (equivalent to the X-ray structure without the ligand) served as the basis for the simulations of apo STARD2 with ER bilayer model. A dihedral restraint was imposed on the $\chi1$ (N, CA, CB, and CG atoms) and $\chi2$ (CA, CB, CG, and CD1 atoms) angles of W101, applying a force of 5 kcal/mol using the extraBonds term of NAMD. These restraints were utilized to maintain the orientation of the W101 sidechain close to that observed in the X-ray structure. Dihedral restraints remained active throughout both the equilibration and production phases of the simulations. All other equilibration and production parameters are similar to those utilized in the holo STARD2-bilayer simulations.

5. Trajectory analysis

The analyses conducted in this study utilized the MDAnalysis package (version 2.4.2)[20,21] to perform various assessments, including root-mean-square deviation (RMSD), minimum distance, Janin plot, and depth of insertion. RMSD analysis focused on the backbone atoms of the protein in comparison to the X-ray structure. Depth of insertion analysis was carried out separately for the last 500 ns of all replicas and then averaged, with the depth calculated as the average distance between each amino acid's Cß atom (excluding glycine) and the average upper phosphate plane.

For interactions analyses, an in-house Python3 code was utilized based on the MDAnalysis package for parsing structure and trajectory files. Hydrogen bond and hydrophobic contact analyses were conducted using the last 500 ns of trajectories. Criteria for hydrogen bonds were set as follows: the distance between the hydrogen atom and the acceptor atom should be 2.4 Å or less, and the angle formed by the hydrogen bond acceptor, hydrogen, and hydrogen bond donor should be 130 degrees or greater. These criteria must be met for at least two consecutive frames and present in all replicas. Hydrophobic contacts were identified when two non-bonded candidate atoms were within a distance of 3 Å or less for a minimum of two consecutive frames and present in all replicas. Cation-π interactions analysis was performed from the binding of the protein to the lipid bilayer until the end of the simulation, with binding defined as a minimum distance between the protein and membrane of less than 2.4 Å, maintained for the duration of the simulation. Cation-π interactions between aromatic rings of aromatic residues and choline head groups were considered to occur when the distances between the aromatic carbon and choline nitrogen were all below 7 Å. Data visualizations were generated using Matplotlib[22], and graphical representations were rendered using ChimeraX software (version 1.7)[23,24].

6. Free energy protocol

The change in free energy within a system as it moves from its initial to final state serves as an illustration of a reversible thermodynamic process, as outlined in the thermodynamic cycle presented in Figure 5 of the main text. We employed an alchemical approach to calculate the contribution of Y84-choline and Y105-choline cation-π interactions to the affinity of the POPC lipid for STARD2. To achieve this goal, we extracted two frames from the first replica of the apo-ER simulation. In the first frame (at 348 ns), no PC lipid has been taken up, consequently, there are no cation-π interactions. However, in the second frame (at 405 ns), a PC lipid has been partially extracted, and the choline moiety of this PC is situated between the sidechains of Y84 and Y105, establishing cation-π interactions with them. We followed an alchemical route for transforming the Y84 and Y105 to alanine, both in the presence and absence of the PC headgroup. We achieved this by performing free energy perturbation (FEP) simulations along the horizontal directions in Figure 5.

The VMD Mutator plugin was used to build the dual topology and hybrid amino acids (Y84A and Y105A). To ensure thermodynamic equilibrium during the transformation from the initial to the final state, we utilized an approach where we annihilated the aromatic side chain and introduced the alanine side chain. This transformation was achieved by gradually scaling the interactions with a parameter lambda (λ) from the initial state (λ=0) to the final state (λ=1). To prevent singularities at smaller values of λ, a shifted soft-core potential was employed. We optimized the protocol to ensure the best convergence of FEP simulations for all mutants. Initially, an energy minimization was conducted on the initial state (λ=0) for 5000 steps. Subsequently, we divided λ into 50 windows for both forward and backward directions for each protein-bilayer system. Each window underwent 0.25 ns of equilibration followed by 0.25 ns of production run, utilizing a time step of

1 fs, resulting in 25 ns of simulations for the complete transformation in each direction. Statistical analyses of the FEP simulations were carried out by combining forward and backward simulations using the Bennett acceptance ratio (BAR)[25] algorithm via the ParseFEP plugin[26] in VMD.

The convergence of FEP simulations was ensured by adhering to "Good Practices"[27] in FEP simulations, which involve stratification, probability distribution overlap, and bidirectional simulations. We utilized a consistent and optimized protocol as described above, which was developed through a stratification approach optimized in our previous work[28]. We verified the overlap of probability distributions for the reference and target states of the system. The energies computed for forward and backward transformations, as well as those derived from the BAR analysis, are presented in Table 2 (main text) for each mutation. The consistency of these energies in both transformations indicates a good overlap of probability distributions (data not shown). Additionally, we checked the stability of the protein-POPC complex and that the cation-π interactions under consideration were retrieved were retrieved in the backward simulations. Figure S12 shows the starting structure, final structure of the forward simulation and last conformation of the backward simulation, illustrating the convergence calculation for the Y84A mutation.

Figure S1. X-ray structures of STARD2. (A) Three X-ray structures of STARD2 in complex with different phosphatidylcholine lipids, shown in cartoon model. The structure of STARD2 in complex with 1,2-dilinoleoyl-sn-glycero-3-phosphocholine (DLPC) (pdb id: 1LN1), 1-palmitoyl-2-linoleoyl-sn-glycero-3-phosphocholine (PLPC) (pdb id: 1LN3), and 1-palmitoyl-2-arachidonoyl-sn-glycero-3-phosphocholine (PAPC) (pdb id: 7U9D) are colored in white, orange, and cyan, respectively. **(B)** The aromatic residues around the choline moiety of phosphatidylcholine in the different X-ray structures are depicted in stick model. Hydrogen atoms are not shown. Oxygen and nitrogen atoms are colored in red and blue, respectively. The color code of carbon atoms follows the color code of panel A.

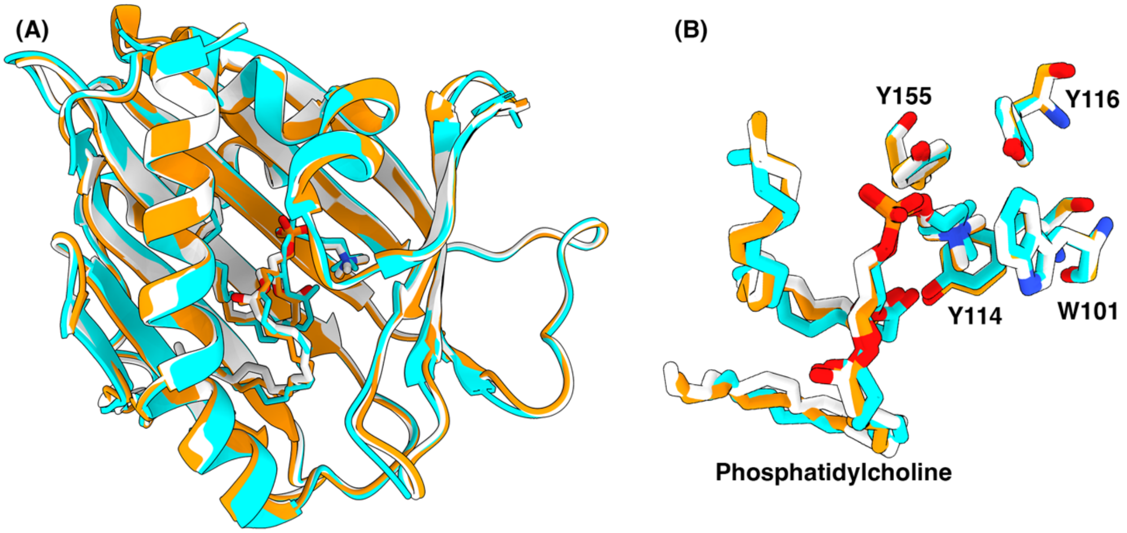

Figure S2. Simulations of apo and holo forms of STARD2 in water: trajectory analysis. (A) Root mean square deviation (RMSD) of protein backbone atoms with respect to X-ray structure along the simulation trajectories. **(B)** Representation of the holo structure (last snapshot of holo_PLPC simulation) and apo structure (last snapshot of apo simulation) in cartoon model. Ω3 loop and C-terminal helix in holo and apo forms are colored in white and red, respectively. **(C)** Orientation of aromatic residues in the aromatic cage in holo and apo structures, colored in white and red, respectively. **(D)** Janin plot representing the χ1 and χ2 angles of W101 in all protein-water simulations.

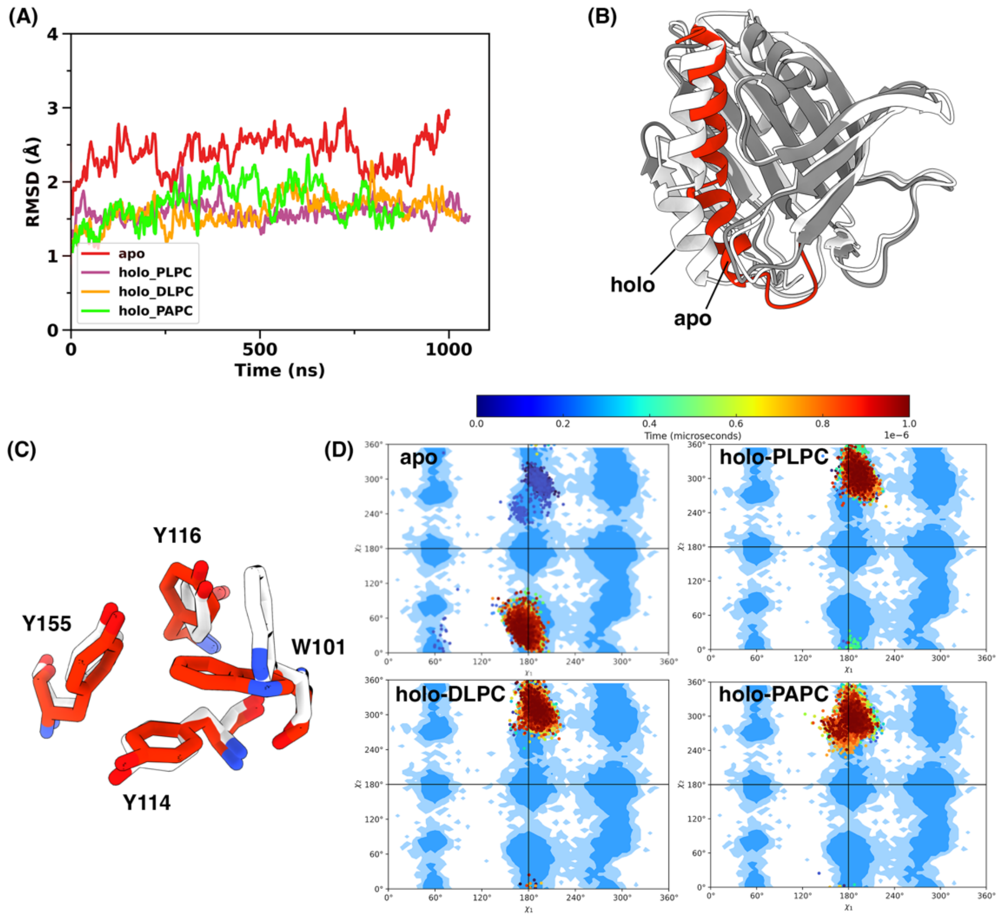

Figure S3. Structures of holo STARD2 from simulations in water. Comparison between structures from the last frame of holo-water simulations (green) to the corresponding X-ray structures (magenta). Protein and cargo lipid are shown in cartoon and stick model, respectively.

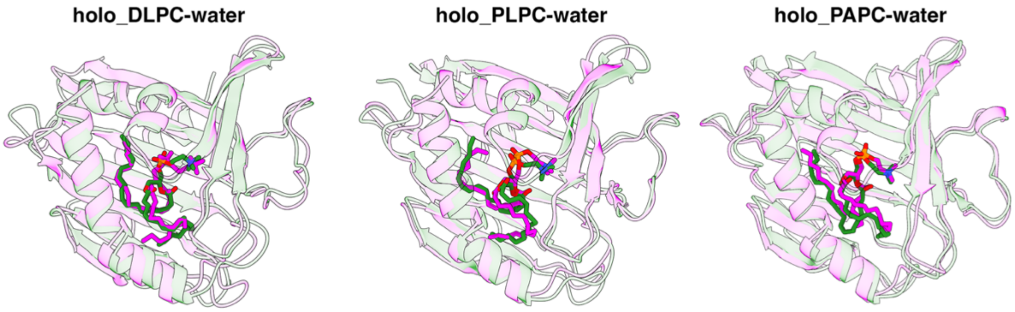

Figure S4. Minimum distance between protein and lipid bilayers along simulation time.

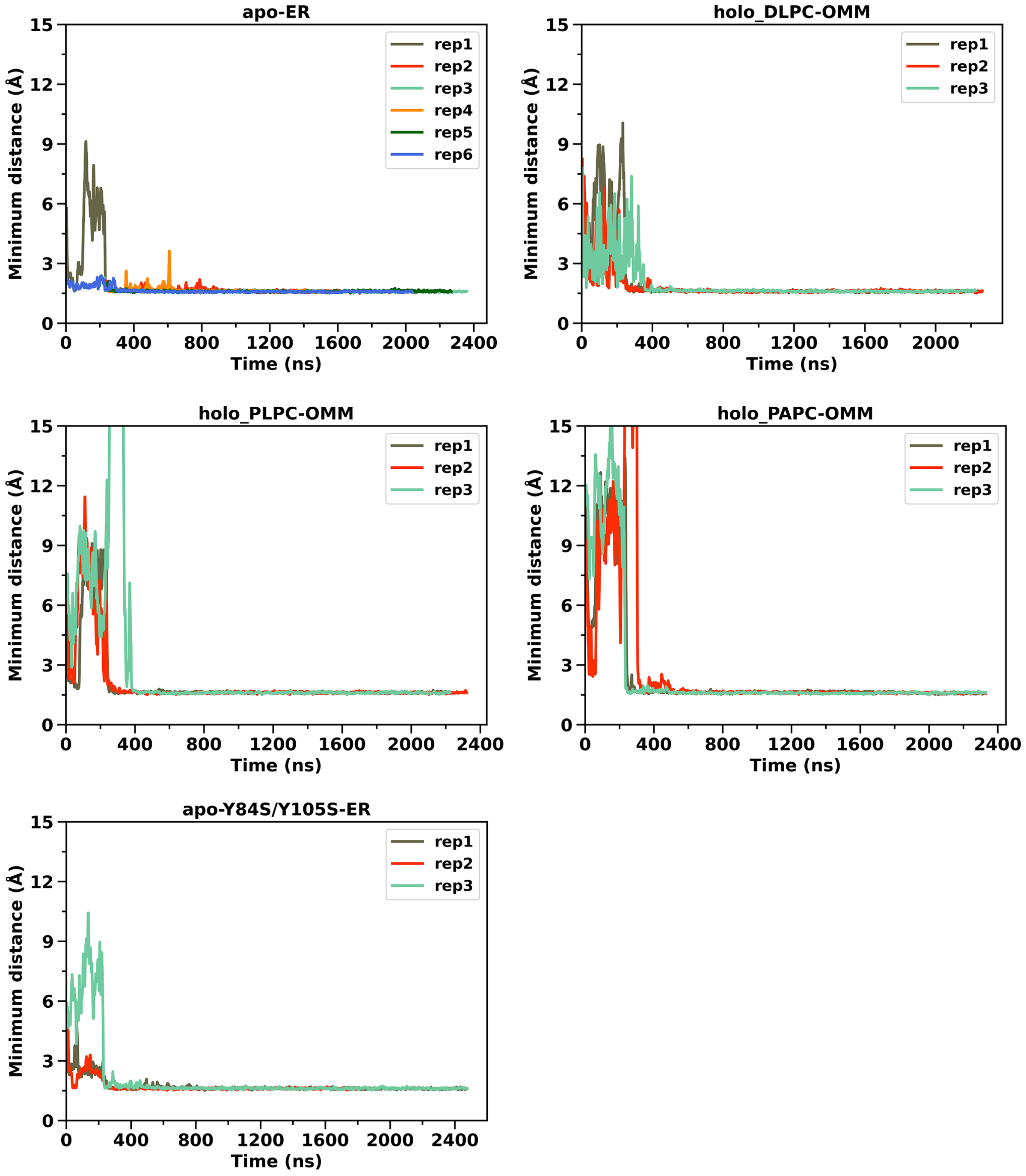

Figure S5. Membrane-bound STARD2: depth of insertion plot for each amino acid. The plotted data are averages over the last 500 ns of each protein-bilayer simulation and plotted for each amino acid. The shaded area represents the standard deviation.

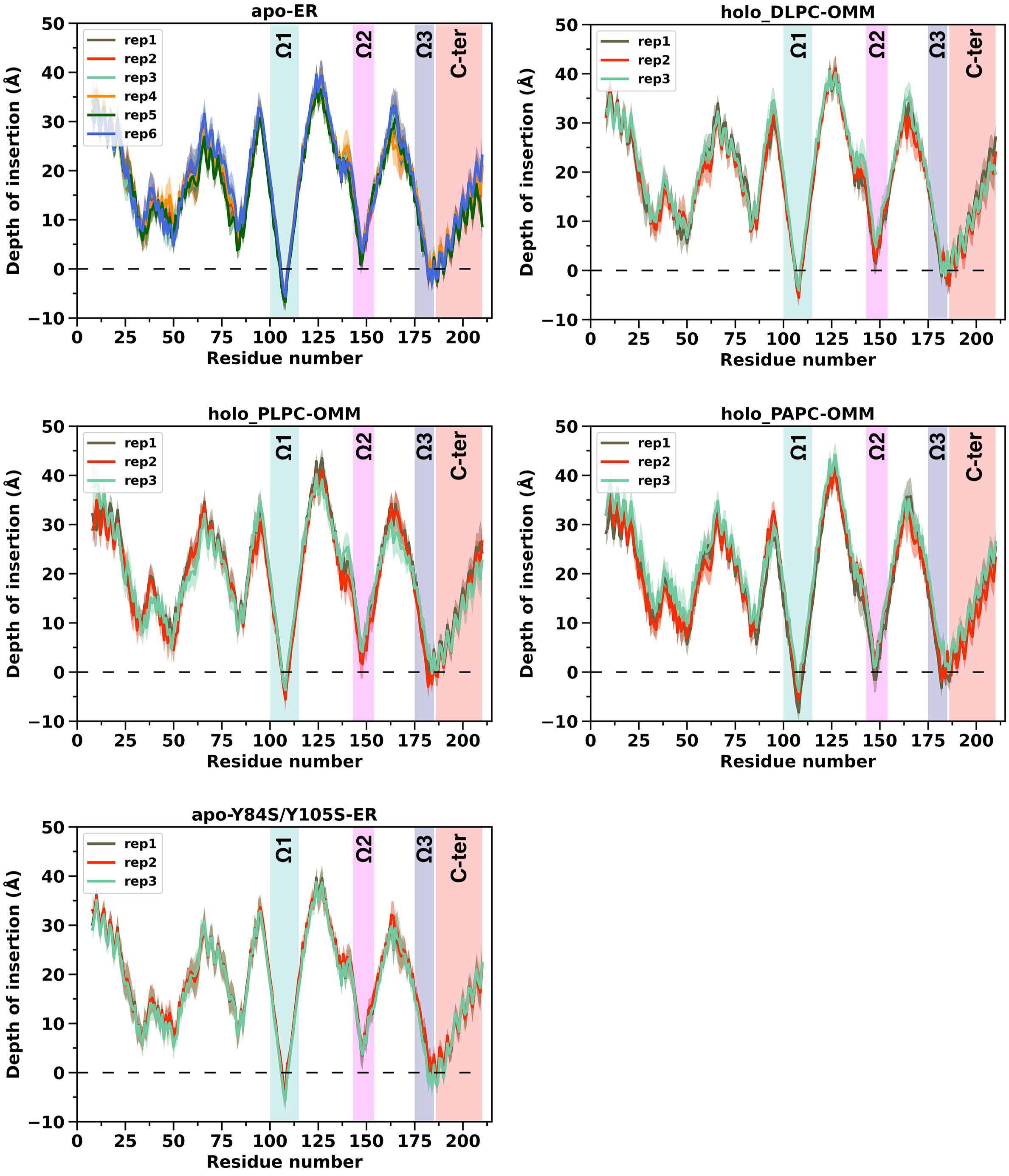

Figure S6. Structures of membrane-bound apo-ER and holo-OMM STARD2. Bound conformations of the apo and holo forms of STARD2 on the ER and OMM models, respectively. Structures are captured in the last frame of the first replica simulation of apo-ER and holo_PLPC-OMM simulations. The protein is depicted in a cartoon model and colored in white, except for the Ω1, Ω2, and Ω4 loops, and the C-terminal helix, which are colored in cyan, magenta, blue, and red, respectively. The membrane is represented in a surface model, colored white with high transparency, to enhance the clarity of the protein-membrane interface.

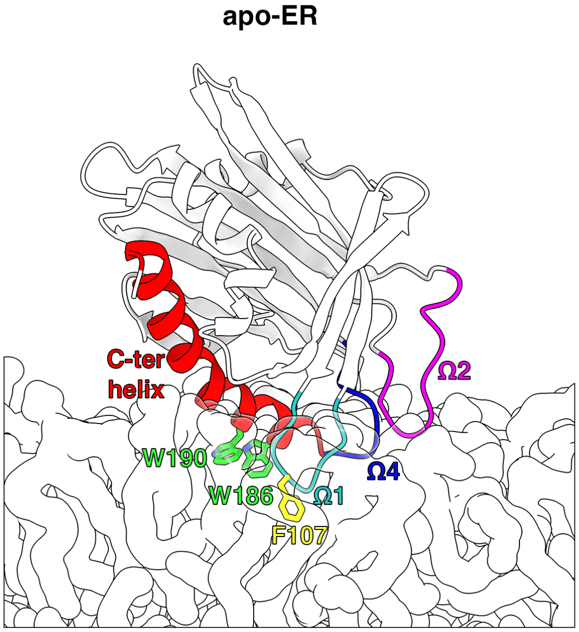

Figure S7. STARD2 holo-OMM simulations: cargo tail insertion into the bilayers. (A) holo_DLPC-OMM, **(B)** holo_PLPC-OMM, and **(C)** holo_PAPC-OMM simulations.

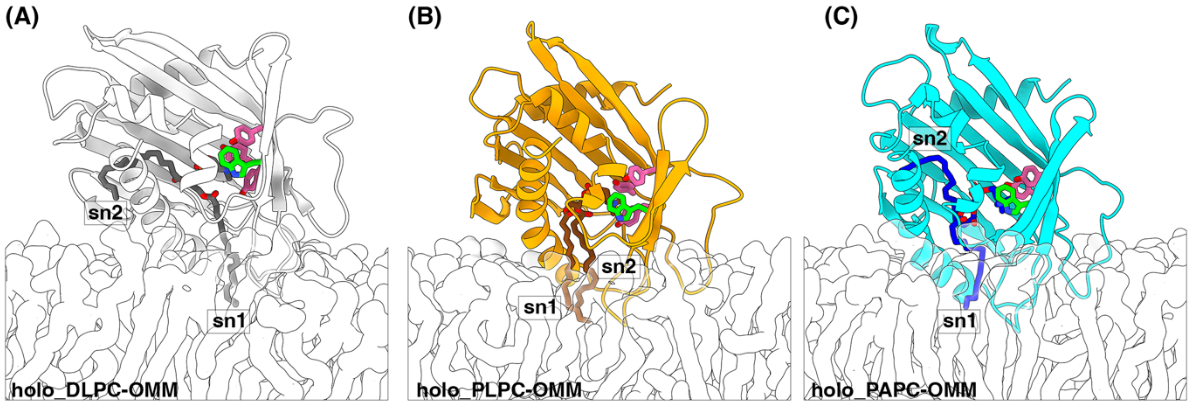

Table S3. Lipids-STARD2 cation-π interactions, between choline groups and aromatic amino acids. Occupancies are given for each of the six replicates (R1-6), together with the average and standard deviation for each amino acid.

Residue	Cation-π interaction occupancy (%)							
	R1	R2	R3	R4	R5	R6	Average	Stdv
Y52	0.3	1.9	7.3	1.2	0.2	0.2	1.9	2.8
W81	20.2	0	0	0	0	0	3.4	8.2
Y84	27.3	10.8	20.3	1.4	28.0	41.0	21.5	14.0
W101	4.4	18.1	49.9	0	0	0	12.1	19.8
Y105	14.3	2.5	11.2	5.6	5.6	29.3	11.4	9.8
F107	1.7	12.7	2.5	0	3.6	3.6	4.0	4.5
Y114	30.9	8.0	9.3	0	0.2	0.2	8.1	11.9
Y116	0.9	17.3	60.5	0	0	0	13.1	24.2
Y155	0.7	4.0	5.9	0	0.1	0	1.8	2.5
W186	16.2	28.7	4.5	29.6	8.5	3.8	15.2	11.7
W190	50.9	17.7	6.8	0	15.1	2.1	15.4	18.7
F199	14.8	2.9	0	0	1.8	2.2	3.6	5.6
Y210	10.0	0	0	0	8.0	0.8	3.1	4.6

Figure S8. Final frame of apo-ER simulation (Rep3) showing the conformations of the extracted POPC from the bilayer. The small inset at the top-right shows the arrangement of aromatic residues (W101, Y114, Y116, and Y155) around the choline moiety of the POPC and highlights residues (Y72, R78, and Q157) forming hydrogen bonds with the phosphate group. Blue dashed lines indicate hydrogen bonds.

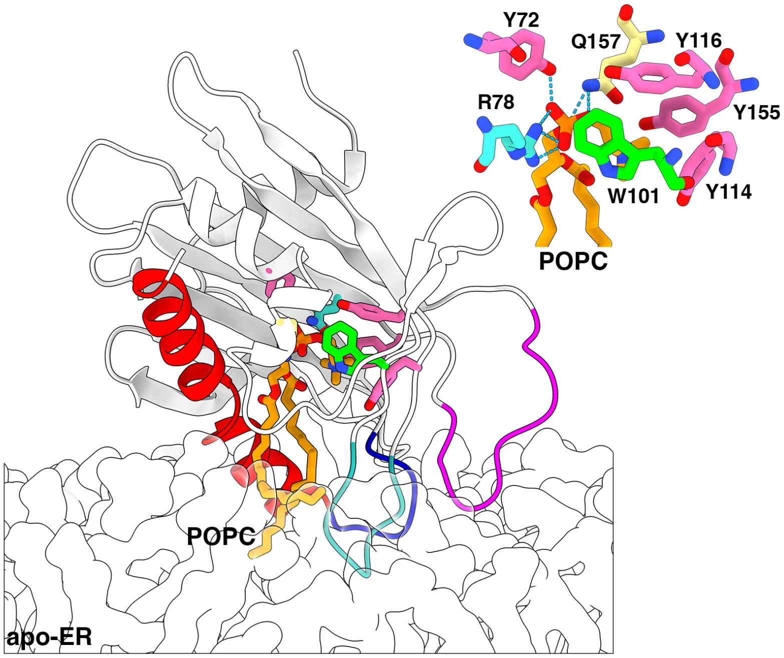

Figure S9. Position of the extracted PC lipid from simulations and X-ray. POPC lipid and amino acids interacting with POPC in the last frame of the apo-ER (Rep3) simulation (shown in orange) compared to their position in the X-ray structure (PDB ID: 1ln1, shown in white).

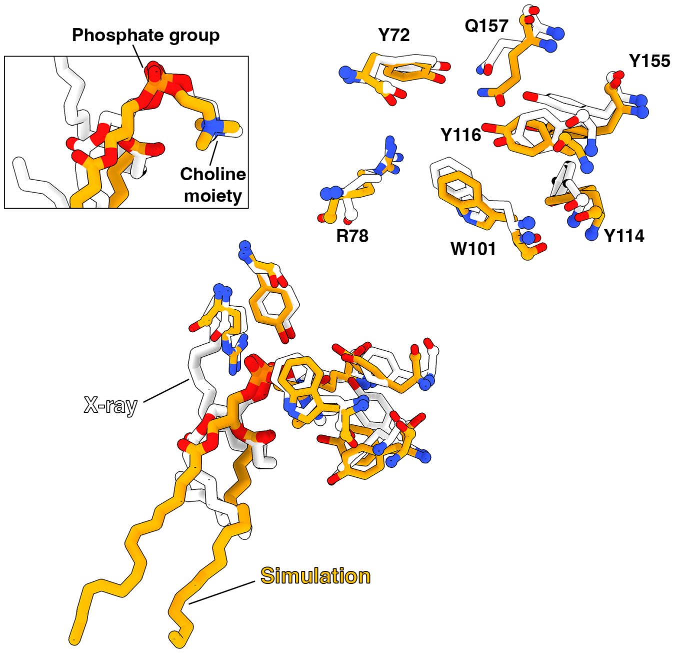

Figure S10. Choline-aromatics cation-π interactions along STARD2-ER simulation replicates for the apo_noRes-ER simulations. (A) The left panels show cation-π interactions between the selected residues at site 1 (W186 and W190), site 2 (Y84 and Y105), and site 3 (W101, Y114, Y116, and Y155) with all membrane lipids, while the right panels focus on cation-π interactions between the same residues and only the POPC bound in the cavity. **(B)** Orientation of the flipped W101 in the absence of restraints, placed in the free space between aromatic residues, thereby preventing the full uptake of the POPC.

(A)

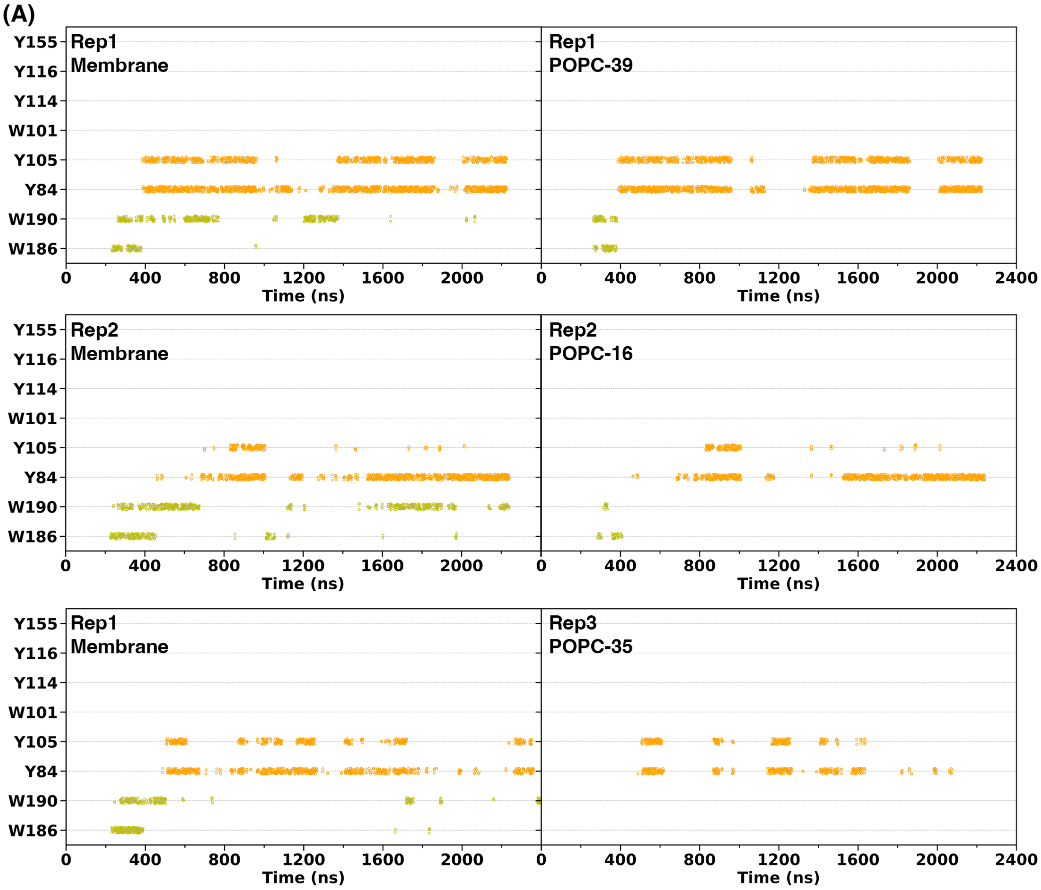

(B)

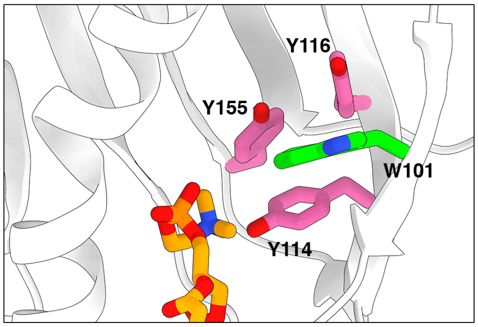

Figure S11. Cation-π interactions between aromatic residues and bilayer lipids. Time-series of cation-π interactions in sites 1-2 in **(A)** apo-ER (Rep6) and **(B)** apo_mut-ER simulations.

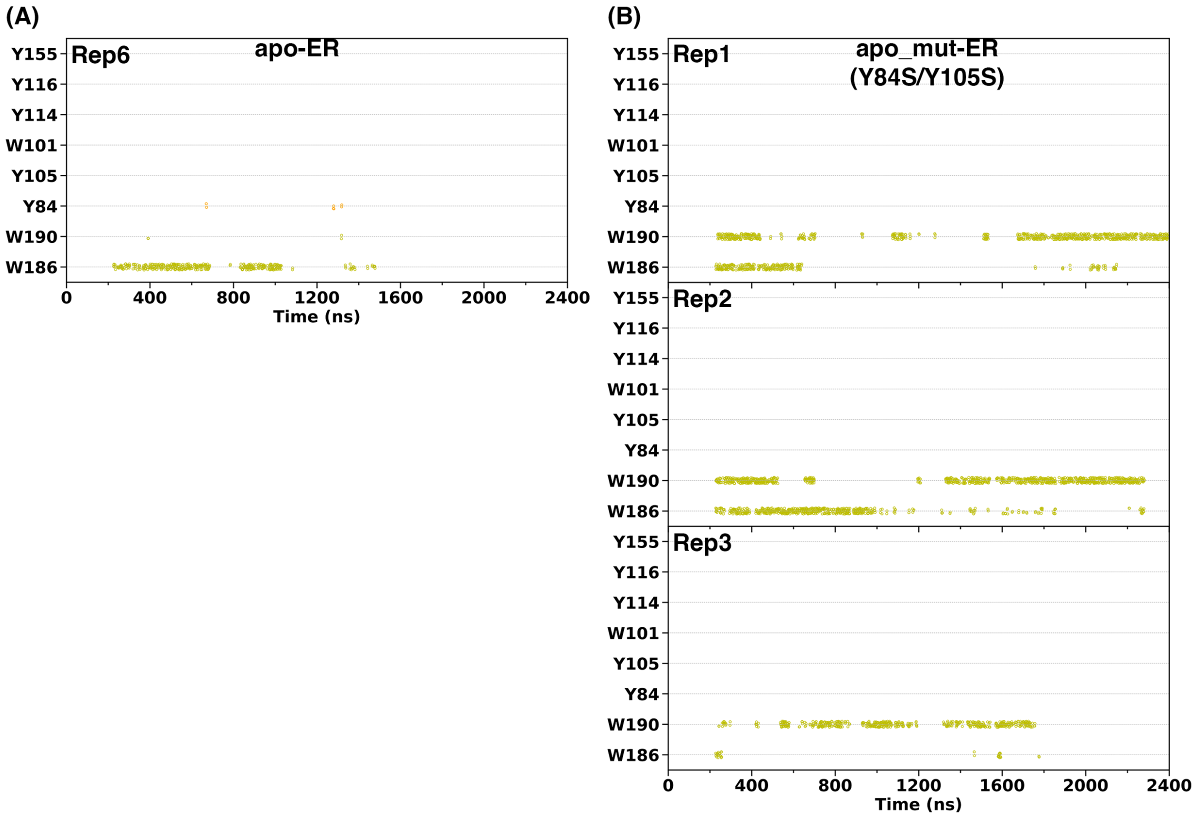

Figure S12. Snapshots from FEP simulations of the Y84A mutation. (A) Initial conformation with the cation-π interaction between Y84 phenol group and the choline headgroup of the lipid molecule. **(B)** Final conformation of the forward simulation where the cation-π interaction is lost due to the substitution of the Y84 by an alanine. **(C)** Last frame from the backward simulation illustrates the regeneration of the cation-π interaction between tyrosine and the choline headgroup.

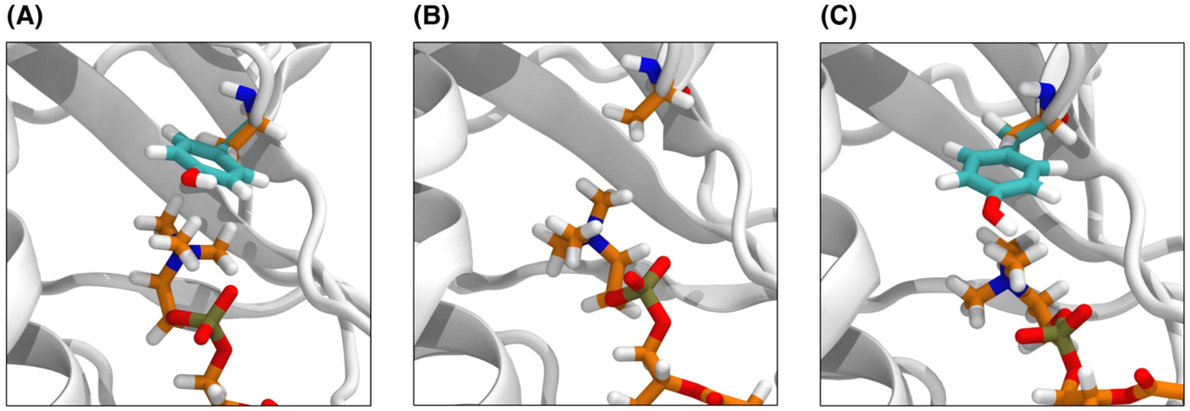

References

(1) Phillips, J. C.; Hardy, D. J.; Maia, J. D. C.; Stone, J. E.; Ribeiro, J. V.; Bernardi, R. C.; Buch, R.; Fiorin, G.; Hénin, J.; Jiang, W.; McGreevy, R.; Melo, M. C. R.; Radak, B. K.; Skeel, R. D.; Singharoy, A.; Wang, Y.; Roux, B.; Aksimentiev, A.; Luthey-Schulten, Z.; Kalé, L. V.; Schulten, K.; Chipot, C.; Tajkhorshid, E. Scalable Molecular Dynamics on CPU and GPU Architectures with NAMD. *J. Chem. Phys.* **2020**, *153* (4), 044130. https://doi.org/10.1063/5.0014475.

(2) Klauda, J. B.; Venable, R. M.; Freites, J. A.; O'Connor, J. W.; Tobias, D. J.; Mondragon-Ramirez, C.; Vorobyov, I.; MacKerell, A. D. Jr.; Pastor, R. W. Update of the CHARMM All-Atom Additive Force Field for Lipids: Validation on Six Lipid Types. *J. Phys. Chem. B* **2010**, *114* (23), 7830–7843. https://doi.org/10.1021/jp101759q.

(3) Best, R. B.; Zhu, X.; Shim, J.; Lopes, P. E. M.; Mittal, J.; Feig, M.; MacKerell, A. D. Jr. Optimization of the Additive CHARMM All-Atom Protein Force Field Targeting Improved Sampling of the Backbone ϕ, ψ and Side-Chain X1 and X2 Dihedral Angles. *J. Chem. Theory Comput.* **2012**, *8* (9), 3257–3273. https://doi.org/10.1021/ct300400x.

(4) Khan, H. M.; MacKerell, A. D. Jr.; Reuter, N. Cation-π Interactions between Methylated Ammonium Groups and Tryptophan in the CHARMM36 Additive Force Field. *J. Chem. Theory Comput.* **2019**, *15* (1), 7–12. https://doi.org/10.1021/acs.jctc.8b00839.

(5) Khan, H. M.; Grauffel, C.; Broer, R.; MacKerell, A. D. Jr.; Havenith, R. W. A.; Reuter, N. Improving the Force Field Description of Tyrosine–Choline Cation–π Interactions: QM Investigation of Phenol–N(Me)4+ Interactions. *J. Chem. Theory Comput.* **2016**, *12* (11), 5585–5595. https://doi.org/10.1021/acs.jctc.6b00654.

(6) Mark, P.; Nilsson, L. Structure and Dynamics of the TIP3P, SPC, and SPC/E Water Models at 298 K. *J. Phys. Chem. A* **2001**, *105* (43), 9954–9960. https://doi.org/10.1021/jp003020w.

(7) Balusek, C.; Hwang, H.; Lau, C. H.; Lundquist, K.; Hazel, A.; Pavlova, A.; Lynch, D. L.; Reggio, P. H.; Wang, Y.; Gumbart, J. C. Accelerating Membrane Simulations with Hydrogen Mass Repartitioning. *J. Chem. Theory Comput.* **2019**, *15* (8), 4673–4686. https://doi.org/10.1021/acs.jctc.9b00160.

(8) Gao, Y.; Lee, J.; Smith, I. P. S.; Lee, H.; Kim, S.; Qi, Y.; Klauda, J. B.; Widmalm, G.; Khalid, S.; Im, W. CHARMM-GUI Supports Hydrogen Mass Repartitioning and Different Protonation States of Phosphates in Lipopolysaccharides. *J. Chem. Inf. Model.* **2021**, *61* (2), 831–839. https://doi.org/10.1021/acs.jcim.0c01360.

(9) Essmann, U.; Perera, L.; Berkowitz, M. L.; Darden, T.; Lee, H.; Pedersen, L. G. A Smooth Particle Mesh Ewald Method. *J. Chem. Phys.* **1995**, *103* (19), 8577–8593. https://doi.org/10.1063/1.470117.

(10) Roderick, S. L.; Chan, W. W.; Agate, D. S.; Olsen, L. R.; Vetting, M. W.; Rajashankar, K. R.; Cohen, D. E. Structure of Human Phosphatidylcholine Transfer Protein in Complex with Its Ligand. *Nat. Struct. Biol.* **2002**, *9* (7), 507–511. https://doi.org/10.1038/nsb812.

(11) Druzak, S. A.; Tardelli, M.; Mays, S. G.; El Bejjani, M.; Mo, X.; Maner-Smith, K. M.; Bowen, T.; Cato, M. L.; Tillman, M. C.; Sugiyama, A.; Xie, Y.; Fu, H.; Cohen, D. E.; Ortlund, E. A. Ligand Dependent Interaction between PC-TP and PPARδ Mitigates Diet-Induced Hepatic Steatosis in Male Mice. *Nat. Commun.* **2023**, *14* (1), 2748. https://doi.org/10.1038/s41467-023-38010-w.

(12) Humphrey, W.; Dalke, A.; Schulten, K. VMD: Visual Molecular Dynamics. *J. Mol. Graph.* **1996**, *14* (1), 33–38. https://doi.org/10.1016/0263-7855(96)00018-5.

(13) Søndergaard, C. R.; Olsson, M. H. M.; Rostkowski, M.; Jensen, J. H. Improved Treatment of Ligands and Coupling Effects in Empirical Calculation and Rationalization of pKa Values. *J. Chem. Theory Comput.* **2011**, *7* (7), 2284–2295. https://doi.org/10.1021/ct200133y.

(14) Jo, S.; Kim, T.; Iyer, V. G.; Im, W. CHARMM-GUI: A Web-Based Graphical User Interface for CHARMM. *J. Comput. Chem.* **2008**, *29* (11), 1859–1865. https://doi.org/10.1002/jcc.20945.

(15) Lee, J.; Cheng, X.; Swails, J. M.; Yeom, M. S.; Eastman, P. K.; Lemkul, J. A.; Wei, S.; Buckner, J.; Jeong, J. C.; Qi, Y.; Jo, S.; Pande, V. S.; Case, D. A.; Brooks, C. L. I.; MacKerell, A. D. Jr.; Klauda, J. B.; Im, W. CHARMM-GUI Input Generator for NAMD, GROMACS, AMBER, OpenMM, and

CHARMM/OpenMM Simulations Using the CHARMM36 Additive Force Field. *J. Chem. Theory Comput.* **2016**, *12* (1), 405–413. https://doi.org/10.1021/acs.jctc.5b00935.

(16) Talandashti, R.; van Ek, L.; Gehin, C.; Xue, D.; Moqadam, M.; Gavin, A.-C.; Reuter, N. Membrane Specificity of the Human Cholesterol Transfer Protein STARD4. *J. Mol. Biol.* **2024**, 168572. https://doi.org/10.1016/j.jmb.2024.168572.

(17) Schenkel, L. C.; Bakovic, M. Formation and Regulation of Mitochondrial Membranes. *Int. J. Cell Biol.* **2014**, *2014*, 709828. https://doi.org/10.1155/2014/709828.

(18) Wu, E. L.; Cheng, X.; Jo, S.; Rui, H.; Song, K. C.; Dávila-Contreras, E. M.; Qi, Y.; Lee, J.; Monje-Galvan, V.; Venable, R. M.; Klauda, J. B.; Im, W. CHARMM-GUI Membrane Builder toward Realistic Biological Membrane Simulations. *J. Comput. Chem.* **2014**, *35* (27), 1997–2004. https://doi.org/10.1002/jcc.23702.

(19) Orabi, E. A.; Öztürk, T. N.; Bernhardt, N.; Faraldo-Gómez, J. D. Corrections in the CHARMM36 Parametrization of Chloride Interactions with Proteins, Lipids, and Alkali Cations, and Extension to Other Halide Anions. *J. Chem. Theory Comput.* **2021**, *17* (10), 6240–6261. https://doi.org/10.1021/acs.jctc.1c00550.

(20) Michaud-Agrawal, N.; Denning, E. J.; Woolf, T. B.; Beckstein, O. MDAnalysis: A Toolkit for the Analysis of Molecular Dynamics Simulations. *J. Comput. Chem.* **2011**, *32* (10), 2319–2327. https://doi.org/10.1002/jcc.21787.

(21) Gowers, R. J.; Linke, M.; Barnoud, J.; Reddy, T. J. E.; Melo, M. N.; Seyler, S. L.; Domański, J.; Dotson, D. L.; Buchoux, S.; Kenney, I. M.; Beckstein, O. MDAnalysis: A Python Package for the Rapid Analysis of Molecular Dynamics Simulations. *Proc. 15th Python Sci. Conf.* **2016**, 98–105. https://doi.org/10.25080/Majora-629e541a-00e.

(22) Caswell, T. A.; Lee, A.; Andrade, E. S. de; Droettboom, M.; Hoffmann, T.; Klymak, J.; Hunter, J.; Firing, E.; Stansby, D.; Varoquaux, N.; Nielsen, J. H.; Root, B.; May, R.; Gustafsson, O.; Elson, P.; Seppänen, J. K.; Lee, J.-J.; Dale, D.; hannah; McDougall, D.; Straw, A.; Hobson, P.; Sunden, K.; Lucas, G.; Gohlke, C.; Vincent, A. F.; Yu, T. S.; Ma, E.; Silvester, S.; Moad, C. Matplotlib/Matplotlib: REL: V3.7.1, 2023. https://doi.org/10.5281/zenodo.7697899.

(23) Pettersen, E. F.; Goddard, T. D.; Huang, C. C.; Meng, E. C.; Couch, G. S.; Croll, T. I.; Morris, J. H.; Ferrin, T. E. UCSF ChimeraX: Structure Visualization for Researchers, Educators, and Developers. *Protein Sci.* **2021**, *30* (1), 70–82. https://doi.org/10.1002/pro.3943.

(24) Meng, E. C.; Goddard, T. D.; Pettersen, E. F.; Couch, G. S.; Pearson, Z. J.; Morris, J. H.; Ferrin, T. E. UCSF ChimeraX: Tools for Structure Building and Analysis. *Protein Sci.* **2023**, *32* (11), e4792. https://doi.org/10.1002/pro.4792.

(25) Bennett, C. H. Efficient Estimation of Free Energy Differences from Monte Carlo Data. *J. Comput. Phys.* **1976**, *22* (2), 245–268. https://doi.org/10.1016/0021-9991(76)90078-4.

(26) Liu, P.; Dehez, F.; Cai, W.; Chipot, C. A Toolkit for the Analysis of Free-Energy Perturbation Calculations. *J. Chem. Theory Comput.* **2012**, *8* (8), 2606–2616. https://doi.org/10.1021/ct300242f.

(27) Pohorille, A.; Jarzynski, C.; Chipot, C. Good Practices in Free-Energy Calculations. *J. Phys. Chem. B* **2010**, *114* (32), 10235–10253. https://doi.org/10.1021/jp102971x.

(28) Waheed, Q.; Khan, H. M.; He, T.; Roberts, M.; Gershenson, A.; Reuter, N. Interfacial Aromatics Mediating Cation−π Interactions with Choline-Containing Lipids Can Contribute as Much to Peripheral Protein Affinity for Membranes as Aromatics Inserted below the Phosphates. *J. Phys. Chem. Lett.* **2019**, *10* (14), 3972–3977. https://doi.org/10.1021/acs.jpclett.9b01639.
