# Supplementary material for: Model Mechanism for Lipid Uptake by the Human STARD2/PC-TP Phosphatidylcholine Transfer Protein
Source: J Phys Chem Lett. 2024 Aug 6;15(32):8287–95. doi: 10.1021/acs.jpclett.4c01743 (PMC11331517; doi:10.1021/acs.jpclett.4c01743)
Supplement: Supplementary file 2 — jz4c01743_si_002.pdf [file jz4c01743_si_002.pdf]

jz-2024-017437.R1

Name: Peer Review Information for "Model Mechanism for Lipid Uptake by the Human STARD2/PC-TP Phosphatidylcholine Transfer Protein"

First Round of Reviewer Comments

Reviewer: 1

Comments to the Author

What is the major advance reported in the paper?

This paper is groundbreaking in that the authors have done extensive simulations to probe the binding of STARD2 to realistic membrane models and investigate the lipid uptake mechanisms. This provides a first look at how this protein can exchange lipids between organelles.

2. What is the immediate significance of this advance?

This is important to the community of researchers in the lipid transport protein area as it is one of the first studies to propose a lipid uptake mechanism for this class of proteins.

3. Technical suggestions

Overall, I found this manuscript well written and an exhaustive study on STARD2 including mutation simulations to test their hypothesis. I believe the manuscript is well suited for publishing in this journal and only have one suggestion that might provide further insight into acyl chain uptake. It is understandable that these simulations were unable to see full acyl chain uptake by this protein. Any thoughts beyond a large conformational change needed for uptake. Could some normal mode analysis based on the simulation trajectories or structural changes after lipid headgroup binding that might provide some potential insight? If initial motions could be extrapolated to provide a more complete mechanism for lipid uptake this would be great.

Reviewer: 2

Comments to the Author

In this study, molecular dynamics (MD) simulations are employed to investigate the uptake and release of lipids by the STARD2 protein. No release events were observed in simulations with a lipid membrane resembling the outer mitochondrial membrane. Instead, more interestingly, partial uptake events were sampled in simulations of the apo form of the protein on a lipid membrane mimicking the composition of the endoplasmic reticulum. Based on these uptake events, the authors propose a three-step mechanism where intermediate steps are stabilized by cation-π interactions between the POPC lipid and protein residues at successive binding sites. These findings are significant, and the manuscript is generally well-written and meticulously organized. The proposed model is supported by independent replicas where similar uptake pathways are observed, enhancing the study's conclusions' robustness.

As a methodological note, I believe the authors should better justify their decision to apply dihedral restraints to residue W101. Since W101 rotates in the apo form compared to the X-ray structure, I would expect the rotated form to be used for simulating lipid uptake. The restraining of W101 is justified under the hypothesis that the configuration of W101 observed in the X-ray structure is a pre-requisite for lipid uptake, but I believe that this hypothesis should be discussed and motivated.

Author's Response to Peer Review Comments:

We are grateful to the two reviewers for their careful reading of our work, their encouraging comments and for their insightful suggestions. Our answers are written below in blue fonts, and we provide a version of the manuscript with our changes highlighted in cyan.

We also used the opportunity of this revision to modify the legend of Figure 4 for the sake of clarity, to update Ref.12 now published in *J.Phys.Chem B*, and to correct Ref.35 as requested.

Reviewer 1:

Recommendation: This paper is publishable subject to minor revisions noted. Further review is not needed.

Comments:
1.What is the major advance reported in the paper?

This paper is groundbreaking in that the authors have done extensive simulations to probe the binding of STARD2 to realistic membrane models and investigate the lipid uptake mechanisms. This provides a first look at how this protein can exchange lipids between organelles.

2. What is the immediate significance of this advance?

This is important to the community of researchers in the lipid transport protein area as it is one of the first studies to propose a lipid uptake mechanism for this class of proteins.

> We truly appreciate the reviewer's positive opinion of our work and thank them for insightful suggestions.

3. Technical suggestions

Overall, I found this manuscript well written and an exhaustive study on STARD2 including mutation simulations to test their hypothesis. I believe the manuscript is well suited for publishing in this journal and only have one suggestion that might provide further insight into acyl chain uptake. It is understandable that these simulations were unable to see full acyl chain uptake by this protein. Any thoughts beyond a large conformational change needed for uptake. Could some normal mode analysis based on the simulation trajectories or structural changes after lipid headgroup binding that might provide some potential insight? If initial motions could be extrapolated to provide a more complete mechanism for lipid uptake this would be great.

> Answer: We agree with the reviewer that understanding the conformational changes of the protein would shed light on the full uptake mechanism. We examined the protein dynamics in the trajectories of the simulations that sampled lipid uptake. To that goal we did a principal components analysis (PCA) of the protein structure using MDAnalysis and ProDy. Unfortunately neither yielded individual PCs that explain a high enough percentage of the variance to provide meaningful answers, and we could not identify clear directions of concerted movements or conformational changes from the analysis. We still agree with the reviewer that PCA or NMA are a promising avenue but it will require a more in-depth analysis to disentangle local from global movements,

and it could be the subject of another study (our trajectories are available for download and for anyone who would be interested in pursuing that route).

Reviewer 2:

Recommendation: This paper is publishable subject to minor revisions noted. Further review is not needed.

Comments:

In this study, molecular dynamics (MD) simulations are employed to investigate the uptake and release of lipids by the STARD2 protein. No release events were observed in simulations with a lipid membrane resembling the outer mitochondrial membrane. Instead, more interestingly, partial uptake events were sampled in simulations of the apo form of the protein on a lipid membrane mimicking the composition of the endoplasmic reticulum. Based on these uptake events, the authors propose a three-step mechanism where intermediate steps are stabilized by cation-π interactions between the POPC lipid and protein residues at successive binding sites. These findings are significant, and the manuscript is generally well-written and meticulously organized. The proposed model is supported by independent replicas where similar uptake pathways are observed, enhancing the study's conclusions' robustness.

Answer: we are delighted to read the reviewer's positive comments on our work and thank them for their useful suggestions.

As a methodological note, I believe the authors should better justify their decision to apply dihedral restraints to residue W101. Since W101 rotates in the apo form compared to the X-ray structure, I would expect the rotated form to be used for simulating lipid uptake. The restraining of W101 is justified under the hypothesis that the configuration of W101 observed in the X-ray structure is a pre-requisite for lipid uptake, but I believe that this hypothesis should be discussed and motivated.

Answer: we agree with the reviewer and have modified the manuscript to (1) better justify the decision to restraint residue W101 during the simulation and (2) report results of simulations in the absence of restraints on W101.

(1) We modified the following sentence in the manuscript:

"To correct for the change of orientation of W101 observed in the apo-water simulation, we applied a dihedral restraint on the W101 $\chi 1$ and $\chi 2$ angles to maintain its experimental conformation and facilitate the lipid uptake."

(2) We had conducted three replicates of the apo-ER simulation without applying any restraints, each ran for 2 µs. This system is called apo_noRes-ER and has now been added to the list of simulated systems in Table S1. The time series of lipid-protein cation-π interactions is provided Figure S10, which is the equivalent of Figure 4 but in the absence of restraints on W101. It shows partial uptake (to sites 1 and 2) but no binding to the distorted aromatic cage (site 3).

We added the following lines in the Results section describing the uptake mechanism:

"Note that we used orientational restraints on W101 in the apo-ER simulations (see Methods section). In the absence of restraints, the flipped W101 conformation led to only partial lipid uptake up to site 2 (see Figure S10) as the indole is placed at the center of the aromatic cage and is not displaced by the POPC headgroup (Fig S10B)."
